# Both cell autonomous and non-autonomous processes modulate the association between replication timing and mutation rate

**DOI:** 10.1038/s41598-023-39463-1

**Published:** 2023-08-12

**Authors:** Oriya Vardi-Yaacov, Adar Yaacov, Shai Rosenberg, Itamar Simon

**Affiliations:** 1 Department of Microbiology and Molecular Genetics, IMRIC, Faculty of Medicine, Hebrew University of Jerusalem, Jerusalem, Israel; 2 Sharett Institute for Oncology, The Gaffin Center for Neuro-Oncology, Hebrew University-Hadassah Medical Center, Jerusalem, Israel; 3 The Wohl Institute for Translational Medicine, Hadassah-Hebrew University Medical Center, Jerusalem, Israel

**Keywords:** Cancer genomics, Tumour immunology, Data processing, Bioinformatics, Gene expression analysis

## Abstract

Cancer somatic mutations are the product of multiple mutational and repair processes, some of which are tightly associated with DNA replication. Mutation rates (MR) are known to be higher in late replication timing (RT) regions, but different processes can affect this association. Systematic analysis of the mutational landscape of 2787 tumors from 32 tumor types revealed that approximately one third of the tumor samples show weak association between replication timing and mutation rate. Further analyses revealed that those samples have unique mutational signatures and are enriched with mutations in genes involved in DNA replication, DNA repair and chromatin structure. Surprisingly, analysis of differentially expressed genes between weak and strong RT-MR association groups revealed that tumors with weak association are enriched with genes associated with cell–cell communication and the immune system, suggesting a non-autonomous response to DNA damage.

## Introduction

The process of DNA replication plays an important role in mutagenesis^1^, with failures leading to the introduction of mismatches and/or to the conversion of DNA damages into mutations. It is therefore not surprising that replication timing (RT), defined as the time in S phase each region is replicated, is strongly associated with mutation rate (MR) in both germline and somatic cells^2,3^. In general, there are many more mutations in regions that replicate in late S phase than in those which replicate in early S phase (reviewed in^4^), suggesting that either mutagenesis, repair, or both occur at different rates in early and late replicating regions.

Mutational signatures are unique combinations of mutations characteristic of various mutagenesis processes^5^. We and others have recently found that the association between replication timing and mutational signatures differs for different cancer mutational processes^6–8^.

A possible approach for finding the mechanisms that distinguish between mutagenesis in early and late replicating regions (ERR and LRR) is to explore mutation rates in cells harboring a mutation in key genes that are components of the DNA replication or repair mechanisms. Indeed, such an approach was successfully carried out by two groups who found that tumors with defects in either mismatch repair (MMR) or global genome nucleotide excision repair (GG-NER) mechanisms^9,10^ do not show higher mutation rates in LRR, suggesting that differences in the efficiency of DNA repair mechanisms are the basis for the difference in mutation rates between ERR and LRR. This conclusion is further supported by analysis of the association of mutation rate and replication timing in xeroderma pigmentosum patients. Patients with severe impairment of the NER lost the association^11^. Interestingly, a recent analysis of the association of replication timing with mutation signatures across 5120 whole-genome sequenced tumors from 40 cancer types did not support this conclusion. Many signatures associated with MMR deficiency (such as SBS6, 14, 15, 20, 21, 26, and 44) show inconsistent association with replication timing^12^.

Replication stress is a hallmark of cancer cells that is associated with increased genomic instability. Replication stress is a double-edged sword for cancer cells. While it promotes tumorigenesis by increasing genomic instability, it also hinders their potential to proliferate by destabilizing replication forks^13^, sensitizes them to chemotherapy and generates neoantigens that expose them to immunotherapy^14–16^. This vulnerability is classically exploited in cancer treatment to increase replication stress to unsustainable levels^14,17^, but new strategies are emerging, exploiting recently identified specificities of the replication stress response. Combination approaches integrating replication stress–inducing agents, such as carboplatin or gemcitabine, with immunotherapies like the immune checkpoint inhibitor nivolumab, have advanced to clinical trials (NCT02944396, NCT03662074, NCT03061188, NCT02734004, NCT02849496, NCT02657889 and NCT02571725).

In addition to cell autonomous effects of DNA damage, it was recently shown that DNA damage and especially replication stress can recruit the immune system to the damaged cell^18^. It has been proposed that DNA damage and replication stress elicit the activation of inflammatory responses that contribute to tumorigenesis in some contexts and to senescence/aging in others^19,20^. Recent studies have found that the two systems can have mutual effects on each other. On the one hand, defects in processing DNA replication stalled forks lead to accumulation of cytosolic DNA and to activation of the cGAS–STING pathway, resulting in the activation of the type I IFN pathway with consequent expression of ISG15 (interferon-stimulated gene 1515)^21^. On the other hand, inflammation itself can cause replication stress. A recent study found that high levels of ISG15, intrinsic or induced by interferon-β, accelerates DNA replication fork progression, resulting in extensive DNA damage and chromosomal aberrations^22^. Despite the growing evidence of association between replication stress and the immune system, a direct link between mutagenesis and the immune system in tumor samples has not yet been shown.

Here we readdressed the question of the contribution of RT to mutational distribution by identifying tumors in which this association is weaker. A systematic analysis of the association between RT and mutation rates using 2787 whole-genome sequenced (WGS) tumors, which are available from the Pan-Cancer Analysis of Whole Genomes (PCAWG^23^), reveals that the association of approximately a third of the samples is much weaker than that of the majority of samples. We hypothesized that analyses of the two groups of tumors, which differ in association between RT and MR, will help us to understand the molecular basis for this association. We show that, as expected, mutational signatures can partially explain the differences between the samples—tumors that contain a large number of mutations associated with mutational signatures that are prevalent in early replication regions have weaker association with RT. Similarly, we found that deleterious mutations in several pathways are enriched in samples showing weak association between RT and MR. Interestingly, analysis of genes differentially expressed between these two groups of tumors revealed involvement of cell–cell communication and of the interaction with immune cells in modulating the effect of RT on mutation rates. These findings go along with recent observations about a link between replication stress and immune response and, as far as we know, this is the first such relationship reported in vivo. Taken together, our comprehensive approach reveals the involvement of both known and novel processes in controlling the genomic distribution of cancer somatic mutations.

## Results

### Using the degree of association between mutation rates and replication timing to characterize tumor samples

**Figure 1:**
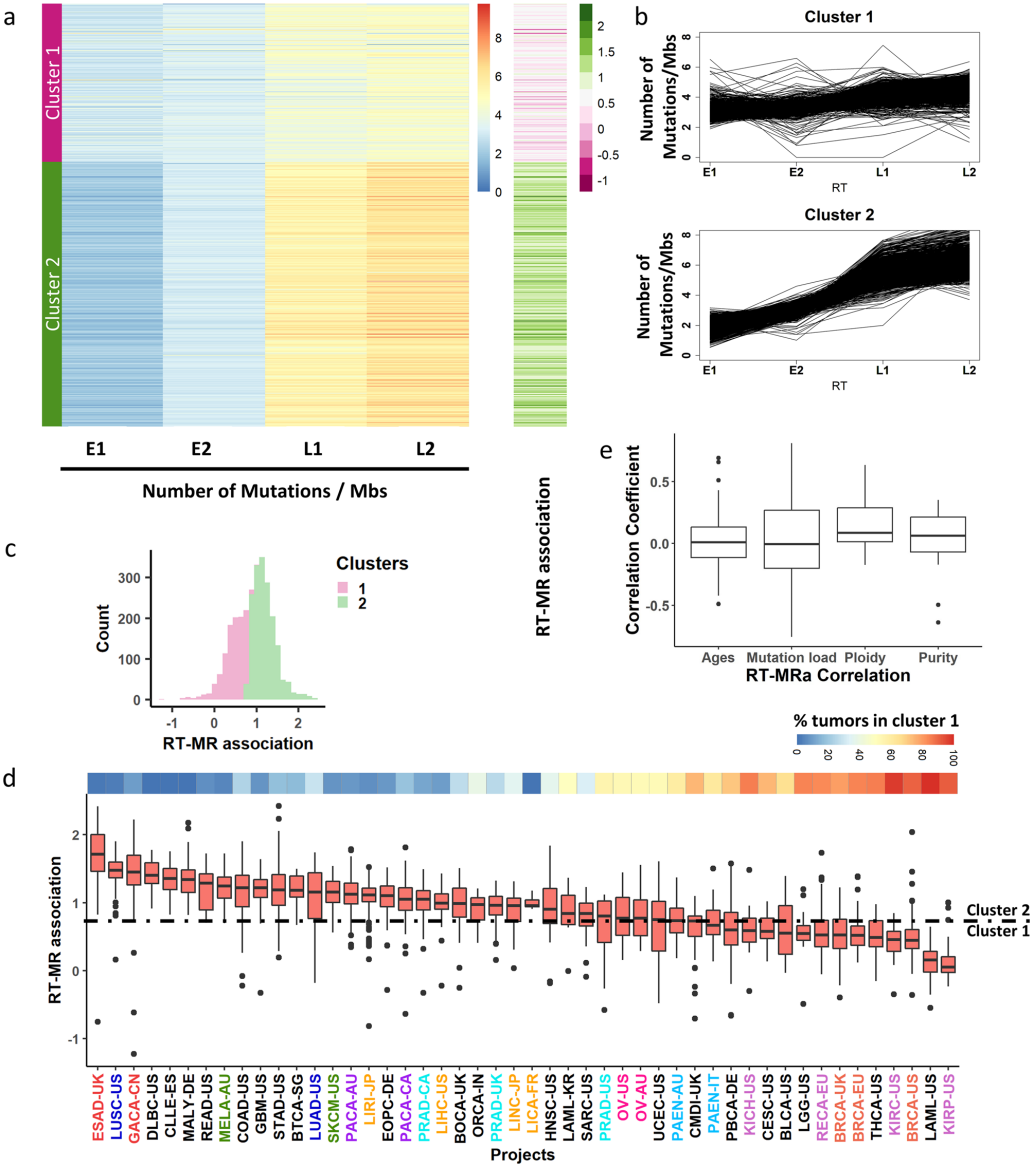
Uneven association between replication timing and mutation rates. (**a**) Heatmap capturing the mutation rate in each sample for four RT bins (E1−L2). The samples were clustered into two distinct clusters (using K-means, see “Methods”) with distinct association between RT and mutation rates. The right heatmap column captures the ratio of the mutation rates between late (L1 + L2) and early (E1 + E2) regions. Similar results were obtained for indel analysis (Supplementary Fig. S1). (**b**) For each cluster, each line shows the mutation rate of a tumor sample at each RT region. (**c**) Histogram of the RT-MRa metric of all tumors is shown. The division into clusters is shown by colors (pink—cluster 1, green—cluster 2). (**d**) Box plot capturing the distribution of the RT-MRa metric in each cancer project. The projects are sorted by the medians of the distributions. Similar cancer types are labeled with the same color. The number of samples in each project is—BLCA-US:23, BOCA-UK:61, BRCA-EU:76, BRCA-UK:44, BRCA-US:92, BTCA-SG:12, CESC-US:20, CLLE-ES:90, CMDI-UK:62, COAD-US:46, DLBC-US:7, EOPC-DE:68, ESAD-UK:97, GACA-CN:32, GBM-US:41, HNSC-US:44, KICH-US:49, KIRC-US:40, KIRP-US:34, LAML-KR:8, LAML-US:33, LGG-US:19, LICA-FR:5, LIHC-US:54, LINC-JP:28, LIRI-JP:260, LUAD-US:42, LUSC-US:48, MALY-DE:100, MELA-AU:70, ORCA-IN:13, OV-AU:69, OV-US:45, PACA-AU:91, PACA-CA:143, PAEN-AU:47, PAEN-IT:34, PBCA-DE:230, PRAD-CA:110, PRAD-UK:78, PRAD-US:20, READ-US:16, RECA-EU:74, SARC-US:34, SKCM-US:38, STAD-US:39, THCA-US:50, UCEC-US:51. The top panel represents the percent of the tumors in cluster 1 in each project. (**e**) Box plot capturing the Pearson correlation coefficient between RT-MRa and age, mutation load, ploidy and purity (see “Methods”).

Mutation rates (MR) are associated with replication timing (RT). As a rule, MR are higher in LRR (late replication regions) and lower in ERR (early replication regions)^4,6^. However, analysis of individual tumors revealed that this association varies considerably, and that there are many tumors with weaker association between MR and RT. In order to systematically investigate this phenomenon, we divided the genome into four equal bins of RT (limiting the analysis to genomic regions with constitutive RT, see “Methods”), and checked the mutation rate in these RT regions for each tumor. Then, we clustered the 2787 WGS tumors into two clusters according to the MR in each bin (“Methods”). Cluster 1 contains 1042 samples in which the association between MR and RT is weak, while cluster 2 contains the remaining 1745 samples in which the association is stronger (Fig. 1a, b). For simplicity of further analyses, we defined the RT-MRa (“RT-MR association”) metric as the log_2_ of the ratio between the MR in the late and the early bins $\left( {{\text{RT}} - {\text{MRa}} = \log_{2} \frac{{{\text{normalized }}\;{\text{mutation}}\;{\text{ count }}\left( {{\text{L}}1 + {\text{L}}2} \right)}}{{{\text{normalized }}\;{\text{mutation}}\;{\text{ count }}\left( {{\text{E}}1 + {\text{E}}2} \right){ }}}} \right)$. This RT-MRa metric ranges from − 1 to 2.5, and most of the samples with RT-MRa score < 0.8 were found in cluster 1 (Fig. 1a (the rightmost column) and c).

Next, we examined the possibility of an association of the RT-MRa metric with tumor type (Fig. 1d). There are cancer types (i.e., different projects) that contain almost only tumors with weak RT-MRa (cluster 1), such as kidney renal papillary cell carcinoma (KIRP) and breast cancer (BRCA), while others are heavily biased toward strong RT-MRa (cluster 2), such as esophageal adenocarcinoma (ESAD) and lung squamous cell carcinoma (LUSC). Furthermore, projects of similar cancer types showed similar behavior in this tendency (Fig. 1d—different projects of similar tumor types were given the same color).

The two clusters may be a consequence of confounding factors such as mutation load or age at diagnosis. In order to explore this possibility, we calculated the correlation between RT-MRa and those features in each project. Both correlations were distributed normally with a mean of 0 (Fig. 1e), suggesting that RT-MRa levels are not associated with mutation rate or age at diagnosis. Interestingly, we did find a small but significant correlation with tumor ploidy level, suggesting that tumors with higher level of duplication tend to have more somatic mutations in late replicating regions (Fig. 1e) Similarly, there is a very small correlation with tumor purity, suggesting that tumors infiltrated by other tissues show lower RT-MRa score (see “Discussion”).

### Mutational signatures association with the RT-MRa metric

In order to explore the factors that influence RT-MRa, we next analyzed the contribution of mutational signatures. The mutational signatures found in each tumor reflect the mutational processes that the tumor underwent. We have shown that mutational signatures differ in their association with RT^6^, and thus they may explain RT-MR association (RT-MRa). We would hypothesize, therefore, that a tumor that mostly underwent mutational processes that are ERR-biased will end up with more mutations in ERR, resulting in a weaker RT-MRa metric as seen in cluster 1.

**Figure 2:**
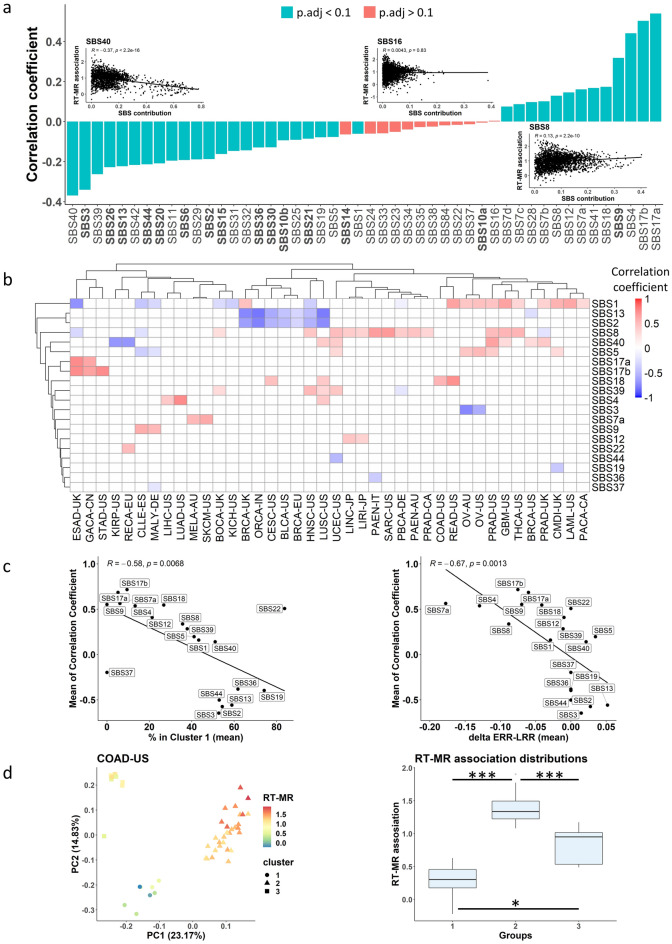
Association between RT-MRa metric and Mutational signatures. (**a**) Pan-cancer analysis of the correlation between RT-MRa metric and signature contributions. Bar chart capturing the correlation coefficient (R) between the RT-MRa score and the relative contribution of each signature. The highlighted signatures indicate signatures with an association with DNA repair defects. Color of bars indicate the statistical significance of the correlation test (adjusted P value < 0.1, FDR-corrected correlation test). Small scatter plots display correlation of RT-MRa metric and signatures contribution for three signatures. (**b**) Project-specific analysis of the correlation between RT-MRa metric and signature contributions. Heatmap capturing the correlation coefficient (R) between the RT-MRa score and the relative contribution of each signature for each project. Only statistically significant associations (adjusted P value < 0.1) are shown. A few examples of signatures with positive or negative correlation between their contribution and RT-MRa are shown in Supplementary Fig. S3. (**c**) Scatter plot of the mean correlation coefficient vs. the mean percentage in cluster 1, taken from Fig. 1D (left panel), and the mean of delta ERR-LRR, taken from^6^ (right panel). Only projects with significant correlation (those shown in **b**) were included in the analysis. (**d**) PCA plots of colon adenocarcinoma (COAD-US) samples separated into three groups based on their mutational signatures’ contribution data. Each dot (representing a tumor sample) is colored according to its RT-MRa score (left panel). Box plots of the RT-MRa distribution of the three different groups. All P values derived from FDR-corrected Wilcoxon rank–sum test. *, P < 0.1; ***, P < 0.001 (right panel).

To explore this relationship between mutational processes and RT, we checked the correlation between the RT-MRa metric and the relative contribution of different mutational signatures. We found mutational signatures that display positive correlation with the RT-MRa metric (i.e., samples with larger contribution of these signatures have stronger RT-MRa or belong to cluster 2), and mutational signatures that display negative correlation (i.e., samples with larger contributions of these signatures have weaker RT-MRa or belong to cluster 1) (Fig. 2a). In some cases, the reason for the associations is clear: signatures that are associated with ERR (such as SBS2 and SBS13^24^) are associated with cluster 1 tumors, whereas signatures associated with LRR (such as SBS17a&b and SBS7a^24^) are associated with cluster 2 tumors. In addition, it is known that defects in DNA repair pathways can cause a different association between RT and MR^9^. Indeed, signatures associated with defects in DNA repair mechanisms (APOBEC, base excision repair, mismatch repair and homologous recombination; Fig. 2a—the bold SBS) display negative correlation with RT-MRa, (i.e., contribute more to cluster 1 tumors). Similar association with signatures associated with DNA repair defects was found using the Kruskal–Wallis rank test (see “Methods” and Supplementary Fig. S2).

Next, we analyzed the association of the RT-MRa metric with mutational signatures for each project separately. This allowed us to identify signatures that are cancer-type specific and thus are less prominent in a pan-cancer analysis. This analysis revealed many signatures that are either positively or negatively associated with the RT-MRa metric in specific projects (Fig. 2b). As expected, signatures with a low mean correlation coefficient value are associated with cluster 1, whereas signatures with a high correlation are associated with cluster 2, which is graphically captured in Fig. 2c (left panel). The association between RT-MRa and mutational signatures is partially explained by the RT bias of each signature^6^. Indeed, plotting the average correlation coefficient as a function of the RT bias (delta ERR-LRR) of each signature, demonstrates this association (Fig. 2c right panel). It should be noted that there are signatures, such as SBS7b and SBS16, that show a strong bias toward ERR^6^, yet are not associated with low RT-MRa (absent in Fig. 2b–d). This is because those signatures have a relatively small contribution to the mutation load of the tumor, and thus their contribution to ERR is eclipsed by other signatures that contribute more mutations to LRR. For example, the UV related signature SBS7b is associated with ERR, yet we do not find it in samples with low RT-MRa, since it is always accompanied by the LRR associated signature SBS7a, which has an average higher contribution (47%) than SBS7b (16%).

After examining each signature separately, we checked whether combinations of signatures contribute to the RT-MRa. To this end, we represented each tumor by a vector containing the relative contribution of each signature, and used principal component analysis (PCA) followed by K-means clustering to define subgroups of tumors within each project (see “Methods”). Our approach revealed that there are distinct subgroups in many projects, and we examined whether these subgroups are associated with the RT-MRa metric using Wilcoxon rank sum test. In many projects, such as COAD-US, a strong association was found (Fig. 2d, Supplementary Fig. S4 and Supplementary Table S1). This finding illustrates the importance and impact of the contribution of signatures to the association between RT and mutation rates.

Taken together, as expected, mutational processes appear to have a strong association with the RT-MRa in many tumors. Yet in many cancer types we found variation in RT-MRa that cannot be explained by signatures (Supplementary Fig. S4 and Table S1).

### Identification of pathways significantly mutated in tumors with weak RT-MR association

**Figure 3:**
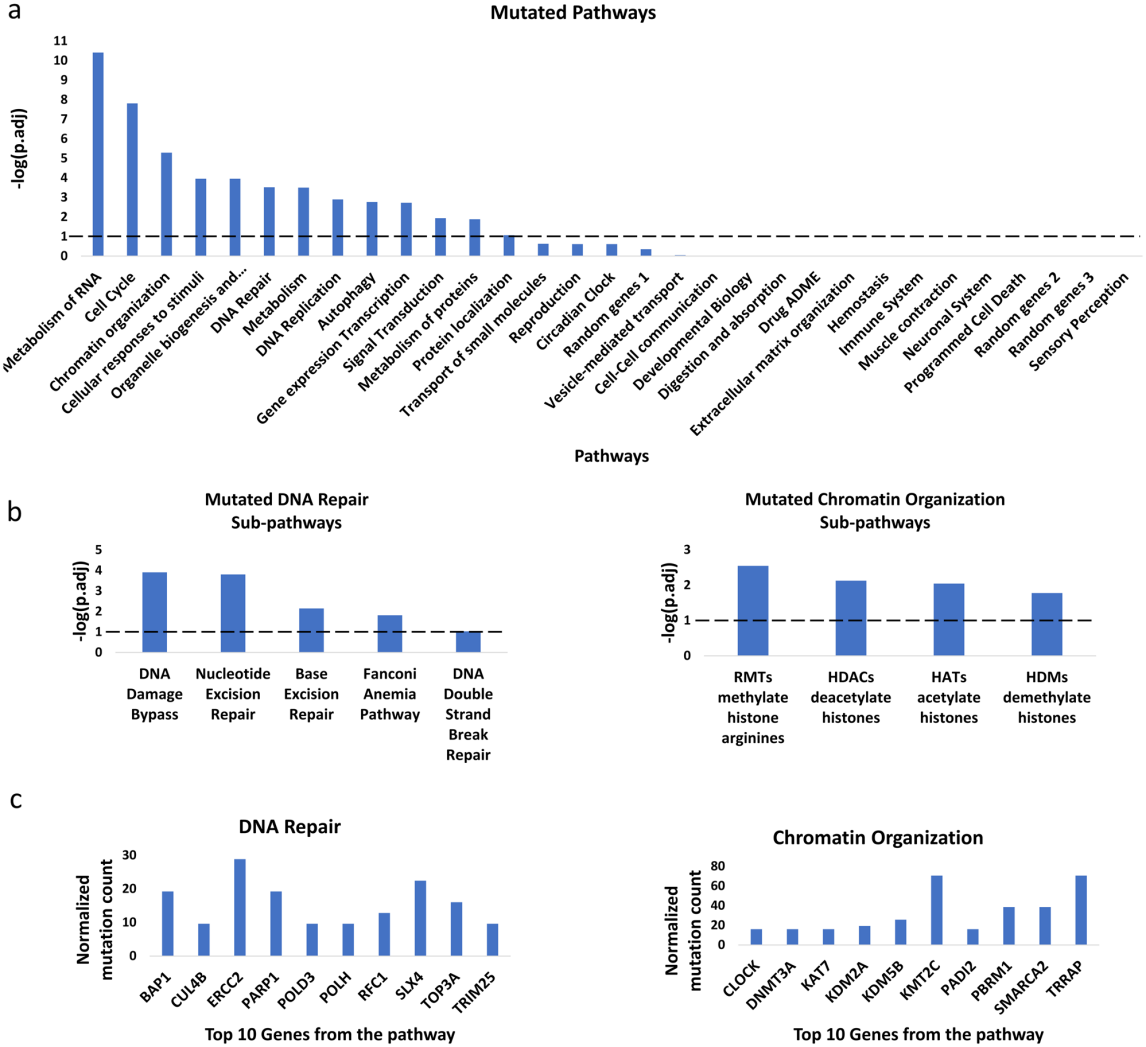
Mutated pathway analysis. (**a**) Testing the different pathways for enrichment of deleterious mutations in the weak RT-MRa group. Binomial tests were performed to test which pathways are enriched. The statistically significant enrichment pathways are those that pass the threshold of adjusted P-value < 0.1 (i.e. − log10(adjusted P-value) > 1, the vertical dashed line). (**b**) The DNA repair and chromatin organization pathways were further divided into sub pathways (using Reactome definition) and the enrichment analyses were repeated. The vertical dashed line designates a threshold of adjusted P-val < 0.1. (**c**) Normalized mutation count for the two groups. For the designated pathways the ten genes with the lowest P-value are shown (see “Methods”; a complete list of enriched genes can be found in Supplementary Table S2).

Next, we explored the possibility that the differences in the RT-MRa scores stem from non-functional genes. To this end, we looked at the abundance of deleterious mutations in tumors with weak and strong RT-MRa (the top 35% and the bottom 35% of scores, respectively). Since deleterious mutations are rare, we performed this analysis at a pathway level, meaning that instead of asking whether a mutation in a particular gene is enriched, we asked if mutations in any gene belonging to a particular pathway are enriched. For the same reason, this analysis was performed in a pan-cancer manner, pooling together samples from all projects. We counted the number of genes with deleterious mutations in all Reactome major pathways^25^and calculated the enrichment in the low RT-MRa group using a binomial test (see “Methods”). As was previously suggested^9–11^, we found that the DNA repair pathway was enriched in the low RT-MRa group (adjusted P value = 3 × 10^–4^; binomial test), suggesting that the difference between the MR in early and late replicating regions is due to differential repair efficiencies. Such differential repair is probably due to differential chromatin structure and indeed mutations in genes associated with chromatin organization were highly enriched as well (adjusted P val = 5 × 10^–6^; binomial test). In addition, we found enrichment of the DNA replication and cell cycle pathways (adjusted P values = 1 × 10^–3^ and 1 × 10^–8^, respectively; binomial test), as well as additional pathways for which their influence on mutation distribution is less clear(Fig. 3a). This conclusion was independent of the definition of the deleterious mutations since similar results were observed using a different definition of effective mutations (Supplementary Fig. S5). Interestingly, dividing the DNA repair pathway into sub-pathways revealed that many of them were enriched (Fig. 3b), suggesting that the association between RT and MR is affected by multiple DNA repair mechanisms and not confined to MMR and GG-NER, as has been previously suggested^9–11^. The ten most enriched genes in DNA repair, DNA replication and chromatin organization pathways are shown (Fig. 3c).

### Identification of differentially expressed genes in tumors with weak RT-MR association

**Figure 4:**
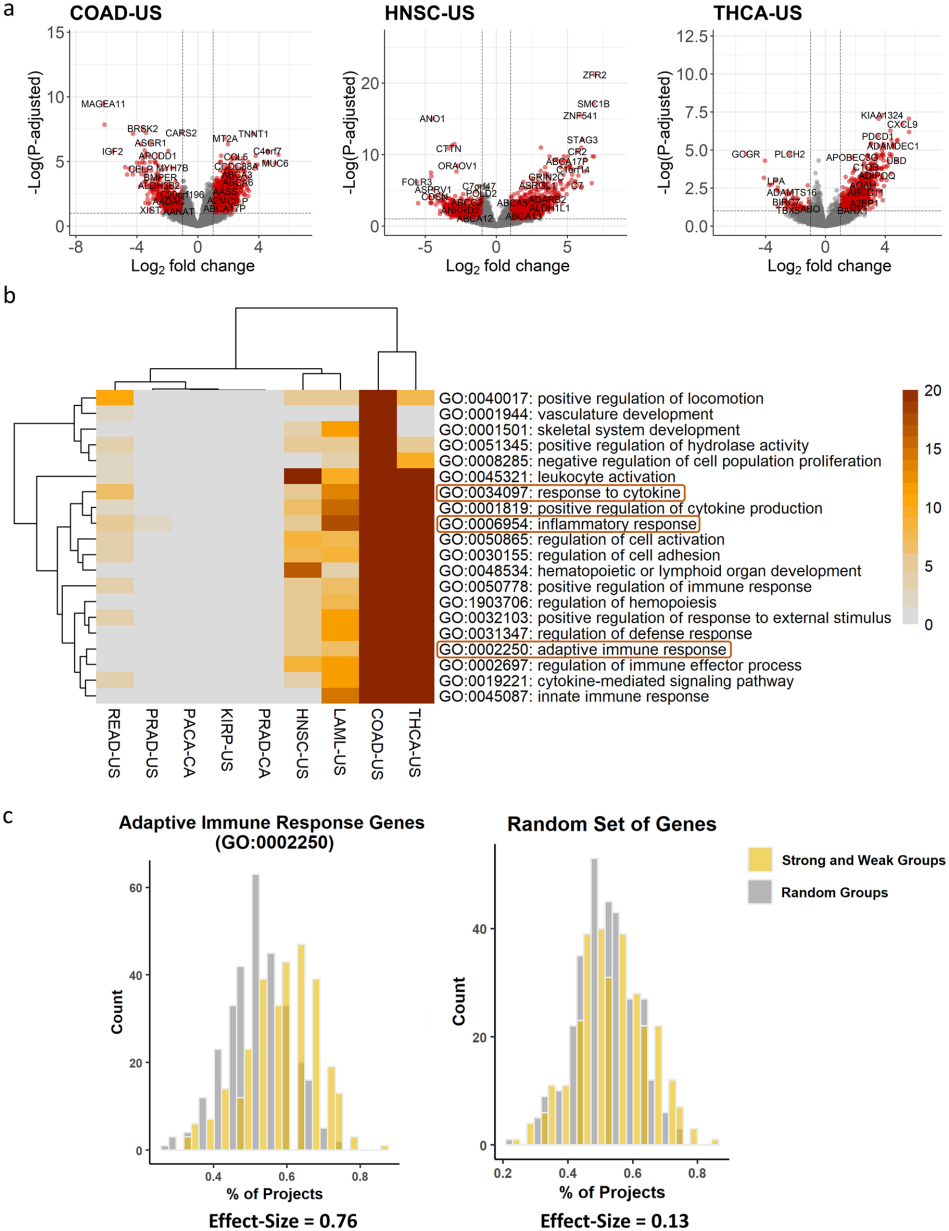
Expression profile analysis. (**a**) Volcano-plots of three different projects. The red points are genes that are differentially expressed significantly between the groups (genes with higher expression in the weak RT-MRa group have log_2_FC > 0). These points pass the thresholds of p.adjusted < 0.1 and |log2FC|> 1. (**b**) Heatmap capturing the significant enrichment of GO categories in the nine selected projects. Non-significant enrichments (p.adjusted < 0.1) are colored grey. (**c**) Histograms capturing the bias in the expression of immune related genes to the weak RT-MRa group in multiple cancer types. For each gene we counted the percentage of projects it was enriched (logFC > 0) in the weak RT-MRa group (orange bars). The distribution is strongly skewed to the right in comparison to a random group of tumors (gray bars) (P val < 10^–16^, effect size = 0.76; paired t-test). Note that this is not the case for a random set of genes (right histograms; P val < 0.007, effect size = 0.13; paired t-test).

In parallel to analyzing the mutated pathways, we performed differential expression analysis to identify correlation of gene expression and pathways with the RT-MRa metric. This analysis was performed on samples for which RNA-seq data exists in addition to the WGS mutation information in ICGC^23^. We analyzed each project separately, since different tissues differ in their expression profiles. As we have shown (Fig. 1d), the RT-MRa metric varies in most projects and thus can be used to define within each project a set of samples with stronger and weaker association between RT and MR. To this end, we divided the samples in each project into three equally sized groups according to the RT-MRa values and used DESeq2 to identify differential expressed genes between the strong RT-MRa and weak RT-MRa groups (Fig. 4a and Supplementary Fig. S6). We found numerous genes in most projects that passed the FDR < 0.1 criterion. In order to control for inflated FDR in DEseq2 analyses^26^, we calculated an experimental FDR by randomizing the tumors in each project, performed differential expression analysis and counted the number of genes that passed the FDR < 0.1 threshold in the randomized data. Only projects in which the number of differentially expressed genes in the randomized data that passed the threshold was less than 10% of the number of genes identified by DESeq2 in the original data were considered valid and were kept for further analyses. Following this analysis, we were left with 10 projects of interest. 9 of these had genes expressed higher in the weak RT-MRa group, and 6 had genes expressed higher in the strong RT-MRa group (5 projects contained genes in both groups). We performed GO annotation analysis on these genes using the Metascape tool^27^, and identified several categories enriched in the weak RT-MRa group. Interestingly, very similar GO terms were enriched in multiple projects (Fig. 4b, Supplementary Tables S3 and S4), suggesting common mechanisms, despite the wide range of tissues and phenotypes. Surprisingly, the reoccurring enriched categories were associated with communications between cells and with the immune system, suggesting that tumors with weak RT-MRa contain a higher degree of infiltration of immune cells (which most probably cause the enrichment of immunological categories). These results were confirmed using a more stringent differential expression identification approach (based on a Wilcoxon test (following^26^)) (Supplementary Fig. S7, Supplementary Tables S5 and S6). In order to confirm this surprising finding, we tried to confirm the generality of the association with the immune system. To this end, we looked at the group of genes that were enriched in several projects. Counting the number of projects each gene was enriched in revealed a large number of genes enriched in multiple projects (Fig. 4c). The 421 differential genes that are enriched in at least 3 projects are also enriched for immunity-associated processes (Supplementary Fig. S8). Furthermore, we were able to show that immune-related genes are expressed higher in the weak RT-MRa group in multiple cancer types (Fig. 4c and Supplementary Fig. S9). By contrast, analyzing genes with higher expression in samples with strong RT-MRa did not reveal any association with cell–cell communication or immunology (Supplementary Fig. S10, Supplementary Tables S7 and S8).

**Figure 5:**
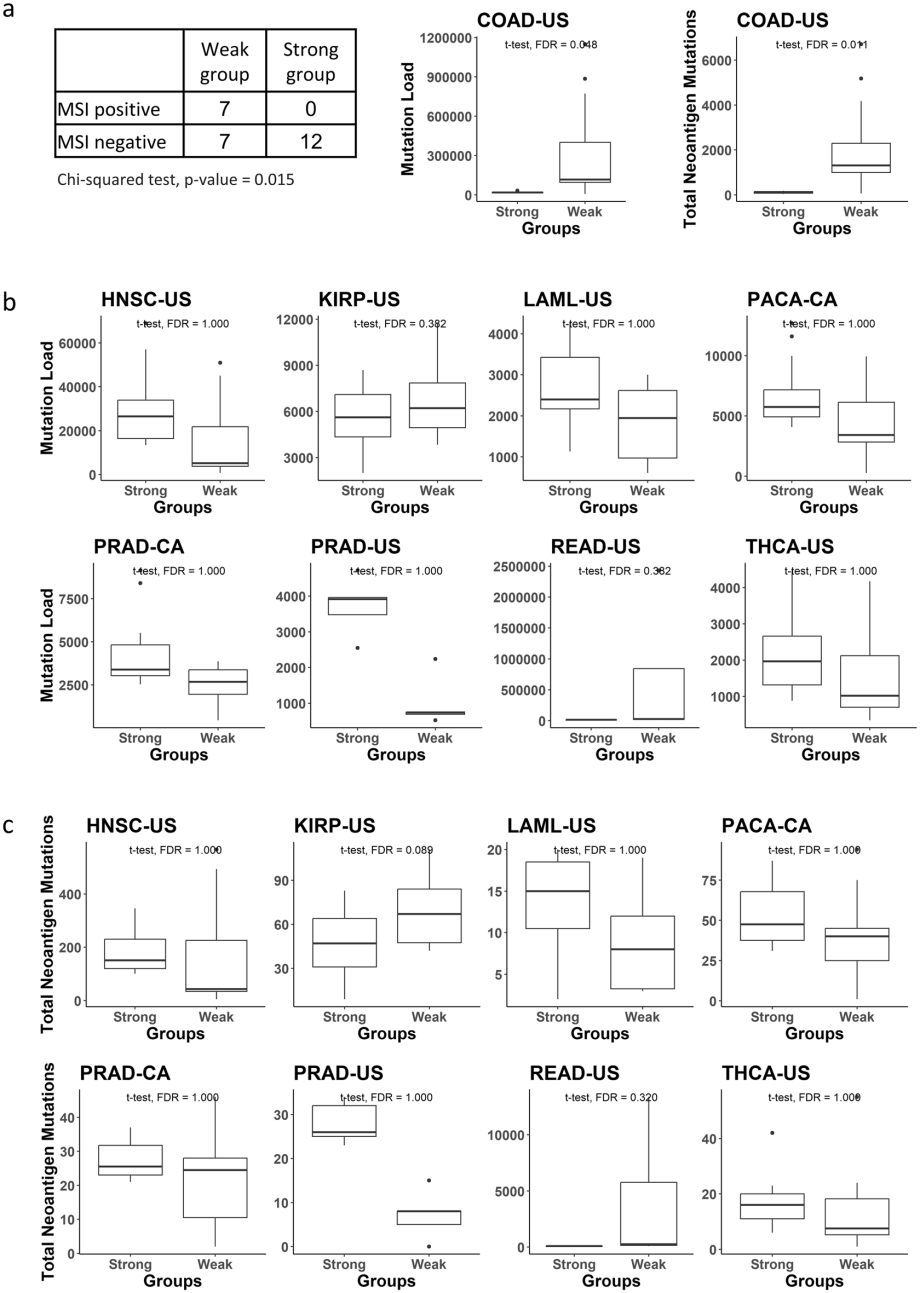
Confounding factors. (**a**) Confounding factors in COAD-US project: Statistic of MSI positive and negative tumors counts in the Strong/Weak groups (left panel), boxplots of the mutation load distribution of the strong and weak groups (middle panel) and of the neoantigen mutations count distribution of the strong and weak groups (right panel). (**b**) Boxplot of the mutation load distribution of the strong and weak groups in the different projects. All adjusted P values derived from one-tailed t-test and Benjamini & Hochberg correction (FDR). (**c**) Boxplot of the neoantigen mutations count distribution of the strong and weak groups in the different projects (see “Methods” for the neoantigen mutations definition). All adjusted P values derived from one-tailed t-test and Benjamini & Hochberg correction (FDR).

The association between RT-MRa and immune genes expression could be either causative or affected by a common confounding factor. It is generally hard to infer causality from associations and thus the only thing that can be done is to look for association between the RT-MRa and other potential confounding factors. Although there was no general association of the RT-MRa metric with either age or mutation load (Fig. 1e), we re-examined those associations in the nine tumor types with genes expressed higher in the weak RT-MRa group. We found that in colon cancer (COAD-US) the weak RT-MRa group is associated with high mutation load and also with neoantigens (see “Methods”). Moreover, for the 26 colon cancer samples, for which we have information about the status of microsatellite instability (MSI)^23^, the weak RT-MRa group is associated with MSI positive tumors (P val = 0.015; Chi square) (Fig. 5a). This suggests that for colon cancer, the association with the immune genes may be a consequence of high mutation load. On the other hand, in all other cases such associations were not found (Fig. 5b, c), suggesting that the involvement of the immune genes may be associated directly with mutation distribution. The associations with age at diagnosis were weak (Supplementary Fig. S11).

## Discussion

Replication timing is strongly associated with mutation rates, with more mutations occurring at LRR (reviewed in^4^). Previous studies suggested that this association stems from more efficient DNA repair in the early replicating, more accessible parts of the genome. Indeed, defects in either mismatch repair (MMR) or global genome nucleotide excision repair (GG-NER) mechanisms abolish the RT-MRa^9–11^. Here we expanded this approach and systematically analyzed the association between RT and MR using 2787 whole-genome sequenced (WGS) tumors (PCAWG;^23^). We found variability in RT-MRa between tumors. In approximately 30% of the tumors the association is weak, with almost the same number of mutations in early and late replicating regions. We grouped the samples according to the degree of their RT-MRa in order to identify the molecular processes that modulate the association.

We found a similar type of variability in RT-MRa for SNVs and for indels (Supplementary Fig. S1), however, when we compared individual samples we found many instances in which the extent of the association was different for SNVs and for indels. This supports the notion that such associations are a result of many factors, explaining why they show variability between individual samples. While the current work concentrates on SNVs, further research is needed for deciphering the factors that contribute to indels and also to CNVs.

Throughout this work we analyzed the association between RT and MR. Yet, we are not excluding the possibility that the actual association is between chromatin accessibility (for which RT is a proxy) and MR. Indeed, calculating a parallel metric, based on data about chromatin accessibility (CA-MRa = chromatin accessibility-MR association metric) gave almost identical results (Supplementary Fig. S12), suggesting that RT and chromatin accessibility are tightly associated and it is very hard to distinguish between them in the genomic context.

We have previously shown that different mutational processes have distinct associations with RT^6^. Thus, we expected that the combination of signatures characterizing each tumor sample would affect the RT-MRa. Indeed, tumors that underwent mutational processes more abundant in ERR show weaker RT-MRa (Fig. 2), reflecting the fact that they have a lot of mutations in ERR. This simple explanation for the variability in RT-MRa explains much of the difference in the RT-MRa score between different tumor types (Figs. 1d and 2c). We also found that mutational signatures related to DNA repair have a greater contribution in tumors with the weaker association, supporting previous observations that defects in DNA repair pathways can affect the association between RT and MR^9–11^. These results differ from the results of a recent pre-print that analyzed the association of cancer mutational signatures with chromatin topographical features including replication timing^12^. They found that signatures associated with MMR deficiency vary in their association with RT between cancer types, whereas our pan cancer analysis revealed much stronger association (bold signatures in Fig. 2a). These differences are probably a consequence of the analysis method. We have analyzed the data in a pan cancer manner while Otlu et al., analyzed each cancer type separately. In addition, we have restricted our analysis to the parts of the genome that replicate at the same time in all examined tissues, whereas Otlu et al., used the entire genome. While each methodology has advantages, we prefer using only the RT constitutive parts of the genome in order to avoid noise introduced by the differences in RT between tissues.

Clustering the samples based on the mutational signatures results in subgroups that differ in their RT-MRa metric (Fig. 2d). Yet, in many cases the signatures are not associated with RT-MRa, suggesting that other processes are modulating the genome-wide distribution of mutations.

Next, we were able to show that deleterious mutations in several pathways are enriched for tumor with weaker RT-MRa. Moreover, this enrichment is not limited to the MMR and GG-NER pathways but extends to additional types of DNA repair mechanisms (Fig. 3b), as well as replication and chromatin structure pathways, suggesting that multiple cellular processes contribute to the uneven distribution of mutations. Interestingly, performing the same analysis on all the Reactome pathways revealed additional pathways which are significantly enriched in deleterious mutation in the low RT-MRa group (Fig. 3a). This suggests that many other processes, including metabolism of RNA and cellular response to stimuli, are somehow involved in the association between RT and MR. Further research is needed in order to decipher the underlying mechanisms for it.

It has been shown that mutations in replication-associated genes (e.g. polymerase proofreading errors) generate more mutations in late replicating regions^28^, nevertheless we found enrichment for mutations in DNA repair genes in the weak RT-MRa group of tumors. This does not exclude the presence of tumors with DNA repair mutations in the high RT-MRa samples which may be the case of mutations in the replicating polymerases. Nevertheless, our analysis only partially supports those previous observations, since only SBS9 (associated with Pol Etta mutations) is positively correlated with the RT-MRa metrric, whereas SBS10a and b (associated with Polymerase epsilon proofreading defects) show no or slightly negative correlation with the RT-MRa metric, respectively (Fig. 2a).

Interestingly, a larger fraction of tumors with high RT-MRa association contain deleterious mutations in DNA repair genes (50%) than in the low RT-MRa group (33%). This finding apparently contradicts the enrichment in DNA repair associated signatures in the low RT-MRa group (Fig. 2a) and the enrichment in the number of deleteriously mutated DNA repair genes in the low RT-MRa group (Fig. 3a). This contradiction together with the recent finding that signatures associated with MMR deficiency vary in their association with RT between cancer types^12^ suggests that mutations in DNA repair genes contribute to perturbation in the RT-MRa but are not always sufficient.

Differential expression analyses revealed that genes highly expressed in the weak RT-MRa samples were enriched in cellular processes involved with interactions between cells and especially with the immune system. Due to the unexpected nature of these results, we took additional measures of precautions. First, we ruled out the possibility of artefacts in the differential expression analysis, by calculating an experimental FDR (based on randomization of the group assignments) and by using a different, more conservative differential expression algorithm (based on a non-parametric test). Secondly, we considered only GO terms that were enriched in several projects (Fig. 4b). Thirdly, we found the same pathways enriched for genes that are differentially expressed in several projects (Supplementary Fig. S8). Finally, we showed that the immune-related categories are enriched in many additional projects (Fig. 4c and Supplementary Fig. S9). We also addressed the possibility that the identified association is due to other confounding factors, by looking at the contribution of mutation load, age at diagnosis and tumor sub-type (Fig. 5 and Supplementary Fig. S11). Finding immune genes highly expressed in samples with weak RT-MRa suggests that these samples have higher degrees of immune cells infiltration. This explanation cannot be valid for the acute myeloid leukemia project (LAML-US) since it is not a solid tumor in which it seems that the immune genes are from the tumor itself. It is important to clarify that that the mutations found in the samples that were used to calculate the RT-MRa score are from the tumor and not from any infiltrating cells, since somatic mutation calling requires multiple occurrences of the same mutation, which is achieved in tumors due to their clonally but not in infiltrating normal cells. This explains why we found immunology genes in the expression profile analysis (Fig. 4) and did not find it in the mutation analysis (Fig. 3).

Interestingly, the finding of immune genes in samples with weak RT-MRa was not associated with the overall RT-MRa level, and it can therefore be found both in colon cancer in which most of the samples have relatively high RT-MRa levels (cluster 2; Fig. 1d) and in thyroid cancer in which most of the samples have relatively low RT-MRa levels (cluster 1; Fig. 1d). This would suggest that the overall level is affected by other factors, including the mutational signatures characteristic of each tumor type, and the immune cells infiltration affects the level of RT-MRa within the general range which is characteristic for each tumor type.

Our findings of a correlation between immune cell infiltration with weak RT-MRa is supported also by the analysis of tumor purity (Fig. 1e), that suggests that tumors that are mixed with other tissues (low purity) are associated with low RT-MRa. It could be explained by two opposite explanations. It is possible that cells with weak RT-MRa recruit the immune system with higher efficiency than those with strong RT-MRa; alternatively, it may be that cell–cell interactions, and particularly interactions of the cancer cells with immune cells, affect the genome-wide distribution of mutations. Indeed, tumors with high mutation loads and especially higher neoantigens load, tend to be more immunogenic^29,30^. This is the case in colon cancer (COAD-US), where we found that the weak RT-MRa group is associated with higher mutation load and with MSI positive tumors (Fig. 5a) suggesting that in this case immune cells infiltration is a consequence of the higher mutation load that is associated with the weak RT-MRa group. On the other hand, we did not find such associations with the other tumor types (Fig. 5b and c), suggesting that either the non-conventional distribution of mutations recruits the immune system, or the involvement of the immune system somehow modulates mutation distribution. Expression profiling reports on the current situation of the sample, while mutation analysis reports on the mutational history of the sample since mutations accumulate over time. Thus, according to the first possibility we interpret the results that the current situation of non-conventional mutation distribution recruits the immune system, whereas according to the latter explanation we ought to assume that the infiltrating immune cells exist also in previous stages of the development of the cancer. Both explanations are consistent with recent findings regarding the association of the immune system with replication stress^13,18–20^. Accumulating evidence indicates that replication stress–inducing agents such as topoisomerase inhibitors and cells deficient in replication stress response genes induce the expression of type I IFNs and pro-inflammatory cytokines^21,31–33^. On the other hand, inflammation causes replication stress, by the influence of ISG15^22^. The fact that there is enrichment of immune genes in the weak RT-MRa samples, raises the question of why is it confined to those samples. This can be explained by the assumption that collision between replication and transcription machineries is the main cause of the immune-related replication stress^34,35^. Such collisions are expected to be found especially in early replicating regions, due to the prevalence of highly expressed genes in these areas^36–39^. This, in turn, leads to weak RT-MRa^40^. Indeed, a higher percentage of mutations fell within genes in the samples with weak RT-MRa, in most (7/9) of the relevant projects (Supplementary Fig. S13).

An association between replication stress and inflammation (by IFI16/STING pathway) has been shown in hidradenitis suppurativa (HS) patients^41^. Yet in the context of cancer, this was demonstrated only in tissue culture systems^13,21,22,31,33^. To the best of our knowledge, this is the first demonstration of the association between mutation distribution and the immune system in patients' tumor samples.

Which parts of the immune system are involved in the identified process? Our results suggest that both the innate and acquired parts of the immune response are involved (Figs. 4b, c and S9), however a more detailed analysis trying to deconvolve the immune cell signal is needed for deeper understanding of the particular contribution of the immune system.

Are infiltrating immune cells always associated with a change in cancer somatic mutation distribution? Although our analysis hints at such a connection, it is not sufficient to support such a strong statement due to the following reasons. First, we were only able to identify reliably differentially expressed genes in 10 cancer types. Secondly, even within this limited group we found enrichment of immune genes only in several projects. Finally, although we ruled out the involvement of neo-antigens in recruiting the immune system in most projects (Fig. 5), it is very hard to rule out the involvement of other confounding factors. Thus, further research is needed in order to confirm and to understand the molecular mechanisms underlying this intriguing finding.

What is the evolutionary advantage of the uneven distribution of mutations along the genome? The regular trend of fewer mutations in the early replicating regions can be understood as a way to guard genes (which are concentrated in the early replicating regions) from mutations and thus its evolutionary advantage is clear. Our finding that in many cancer samples the distribution of mutations is almost even between early and late replicating regions raises a question about its advantages. It may be that it does not give advantage and is merely consequence of factors that affect mutation distribution. Yet, it is plausible to speculate that it may be advantageous in recruiting the immune systems to those cells. Thus it may be that actually, the low RT-MRa pattern is more abundant but cells with higher mutation rate in ERR specifically recruit the immune system and get eliminated before developing into a mature cancer. This intriguing speculation should be tested by performing a similar analysis in normal cells for somatic mutations^42^.

## Methods

### Data sources

We downloaded somatic mutation calls (VCF files) from the PCAWG consortium release of 2,787 whole-cancer genomes across 38 tumor types^23^. The data consists of two sources—The International Cancer Genome Consortium (ICGC; 1902 samples) and The Cancer Genome Atlas (TCGA; 885 samples). Each source utilized its standard variant call pipeline (Consensus calls for ICGC, and the Broad Institute variant calling pipeline for TCGA). The somatic mutation profile of the two consortiums were very similar both in terms of 96 trinucleotide context, and in terms of mutational signatures^6^. Accordingly, we combined the mutation calls data for all analyses.

In addition, we downloaded expression data available for 1401 of the WGS samples in ICGC. We only included data from primary tumors for the differential expression analysis.

### Cluster division

To minimize the effect of variation in RT between cell types, we only used the constitutive RT regions for our analyses, which constitute approximately 40% of the human genome that have the same RT in 26 tissues examined^43^ and are also similar in cancer^6^. Constitutive RT regions were divided into 4 equal bins each spanning the same range of RT: earliest, intermediate early, intermediate late, latest (E1, E2, L1, L2 respectively). Among the constitutive RT regions—498.6 Mb are defined as E1; 227.6 Mb are defined as E2; 238.4 Mb are defined as L1; and 324.6 Mb as L2. For each tumor, the number of mutations in each of the four regions was counted. These counts were normalized for the regions' sizes and the number of mutations per 1 Mb were kept. Thus, each tumor is characterized by a vector of length four, which we used for k-means clustering (Spearman rank correlation method) that divided the data into two different clusters. The RT-MRa (“RT-MR association”) metric is $RT - MRa = \log_{2} \frac{{{\text{normalized mutation count }}\left( {{\text{L}}1 + {\text{L}}2} \right)}}{{{\text{normalized mutation count }}\left( {{\text{E}}1 + {\text{E}}2} \right)}}$.

### The association between the confounding factors and RT-MRa

To analyze the effect of some confounding factors on the RT-MRa measurement we downloaded data of ages, ploidy and purity of the tumors from the PCAWG consortium^23^ and calculated the mutation load for each tumor. For each cancer type we calculated the correlation coefficient between the RT-MRa of the tumors and the specific factor. We show the distribution of the correlation coefficient measurements in all cancer types in Fig. 1e.

### The association between the signature contribution and clusters tumors

The data of the mutational signatures and their association to RT were taken from Yaacov et al.^6^. For each mutational signature we performed correlation test (by the cor.test function in R) with the RT-MRa metric. This was done both for a pool of all tumors (pan-cancer analyses) and for individual projects separately. In the pan-cancer analysis, the test was performed on the tumors in which the signature contribution was above zero. In the cancer-specific analysis, the test was performed on the condition that the mean signature contribution in the project's tumors was above 5%. The FDR correction was done by the p.adjust (Benjamini–Hochberg Procedure) function in R.

Pan-cancer analyses were also done using ranking statistics. For each mutational signature the tumors were sorted according to the relative contribution of the signature to the overall mutation load. We excluded tumors for which the contribution of the specific signature was below 5%. The association of the rank versus the cluster annotation was assessed using the Kruskal–Wallis rank test. Only statistically significant signatures were shown in Supplementary Fig. S2.

For examining the contribution of combinations of signatures to the RT-MRa we represented each tumor sample by the vector of the contribution of each mutational signature to it and used principal components analysis (PCA) to identify groups of similar samples. The resulting groups were further separated by K-means clustering (using the kmeans function in R) to determine the number of clusters by the maximum number of average silhouette widths of the clusters (using the factoextra::fviz_nbclust R function). We examined the association between our RT-MRa metric and the different groups by Wilcoxon rank-sum test for each pair of clusters. Finally, we computed and plotted the PCA using the stats::prcomp and ggplot2::autoplot functions, using the clusters we found.

### Mutational analysis

We focused on all the Reactome pathways^25^. As an additional control we also performed the analysis three times with 500 random chosen genes.. For the DNA repair and chromatin organization pathways we also analyzed sub pathways (defined by the Reactome).

To focus only on mutations that have a deleterious effect on the protein we used vcf2maf software^44^, which provide three different predictions based on Ensembl Variant Effect Predictor (VEP)^45^, the Sorting Intolerant from Tolerant (SIFT) algorithm^46^ and PolyPhen (Polymorphism Phenotyping)^47^.

In order to analyze the most different tumors from the two clusters we only focused on the top and bottom 35% RT-MRa score tumors.

For each pathway, deleterious mutated genes in each group were counted, and then we performed a one-sided binomial test to examine which mutated pathway is enriched in the weak RT-MRa group. The null hypothesis is that a similar ratio of deleterious mutations between the groups should be found in all pathways. Thus, we used the ratio in the total deleterious mutations in the strong and weak groups as the P in the binomial test. In all cases we corrected for multiple hypotheses testing by FDR. Sub pathway analyses were performed in the same way.

We performed the same statistical test for all individual genes (Supplementary Table S2). The 10 genes with the lowest P value are shown in Fig. 3C. The mutations in each gene in each group were counted and normalized to 100 K mutations.

For counting the neoantigen mutations in the tumors (Fig. 5) we included only mutations in genes that follow these definitions in the vcf2maf results: Splice Site, Nonsense Mutation, Frame Shift Del, Frame Shift Ins, Nonstop Mutation, Translation Start Site, In Frame Ins, In Frame Del, Missense Mutation, Splice Region.

### Differential expression analysis

The differential expression analysis was performed for each cancer project separately. Each project is divided into 3 equal groups according to the RT-MRa score. Then, we used the available expression data from the ICGC to examine which genes are expressed differently between the two extreme groups either by DEseq2^48^ or by Wilcoxon rank-sum test^26^. The Wilcoxon test was performed on a preprocessed count matrix using the edgeR package^49^. The obtained P values of both methods were corrected for multiple hypotheses using FDR. In addition, for the DEseq2 results, we calculated an experimental FDR by randomizing the tumors in each group in each project, re-identifying differentially expressed genes and counting the number of genes that passed the FDR < 0.1 criteria in the randomized data.

To examine the bias in the expression of immune-related genes in the weak RT-MRa group in multiple cancer types (Fig. 4C) we calculated the average expression of genes from immune-related GO terms (and from control GO terms) in the weak and strong RT-MRa groups. Then we calculated the percentage of projects with higher expression (logFC > 0) in the weak RT-MRa group. We performed the same analysis on the results of DESeq2 run on 2 random groups of tumors and performed a paired t-test on both lists. From this result we calculated the effect size using this formula: $d = t\sqrt {\frac{{n_{1} + n_{2} }}{{n_{1} \cdot n_{2} }}}$

### GO annotation analysis

For each project we took the genes that were differentially expressed in one of the groups (strong or weak) separately and analyzed it for GO pathways enrichment using Metascape^27^. Using all human genes as the background. We limited our analyses to “GO Biological Processes (1148)”. We used the “Heatmap of enriched terms across input gene lists” from the result page. The identification of genes differentially expressed in multiple projects was done using the “Evidence.csv” file (Supplementary Tables S3, S5 and S7). The genes enriched in multiple projects were rerun in Metascape and used the “Heatmap of selected GO”. In addition, we provided the “FINAL_GO.csv” file (Supplementary Tables S4, S6 and S8).

### Chromatin annotations

We downloaded chromatin annotation data from a recent publication^50^. Regions annotated as either “active” or “active2” were defined as active chromatin whereas the “inactive” regions were defined as inactive chromatin. For each tumor, the number of mutations in each of the two regions was counted. These counts were normalized for the region's sizes and the number of mutations per 1 Mb were kept. The Chromatin accessibility—MRa (“CA-MR association”) metric is

$$CA - MRa = \log_{2} \frac{{{\text{normalized mutation count }}\left( {{\mathbf{Inactive}}{\text{ regions}}} \right)}}{{{\text{normalized mutation count }}\left( {{\mathbf{Active}}{\text{ regions}}} \right){ }}}.$$

Finally, we calculated the correlation between RT-MRa and Chromatin-MRa across the tumors.

### Statistics

Statistical analyses were performed using R version 4.1.2. For multiple comparisons, P values were corrected by FDR using the p.adjust(method = “BH"”) function in R. Plots were generated using ggplot2, ggpubr, and pheatmap R packages.

### Supplementary Information

Supplementary Legends.

Supplementary Figures.

Supplementary Table S1.

Supplementary Table S2.

Supplementary Table S3.

Supplementary Table S4.

Supplementary Table S5.

Supplementary Table S6.

Supplementary Table S7.

Supplementary Table S8.

## Data Availability

The datasets analyzed during the current study are available in the PCAWG consortium repository [https://dcc.icgc.org/].
