# Supplementary material for: Both cell autonomous and non-autonomous processes modulate the association between replication timing and mutation rate
Source: Sci Rep. 2023 Aug 12;13:13143. doi: 10.1038/s41598-023-39463-1 (PMC10423235; doi:10.1038/s41598-023-39463-1)
Supplement: Supplementary file 1 — Supplementary Legends. [file 41598_2023_39463_MOESM1_ESM.docx]

**Supplementary Figures**

**Supplementary Figure S1**


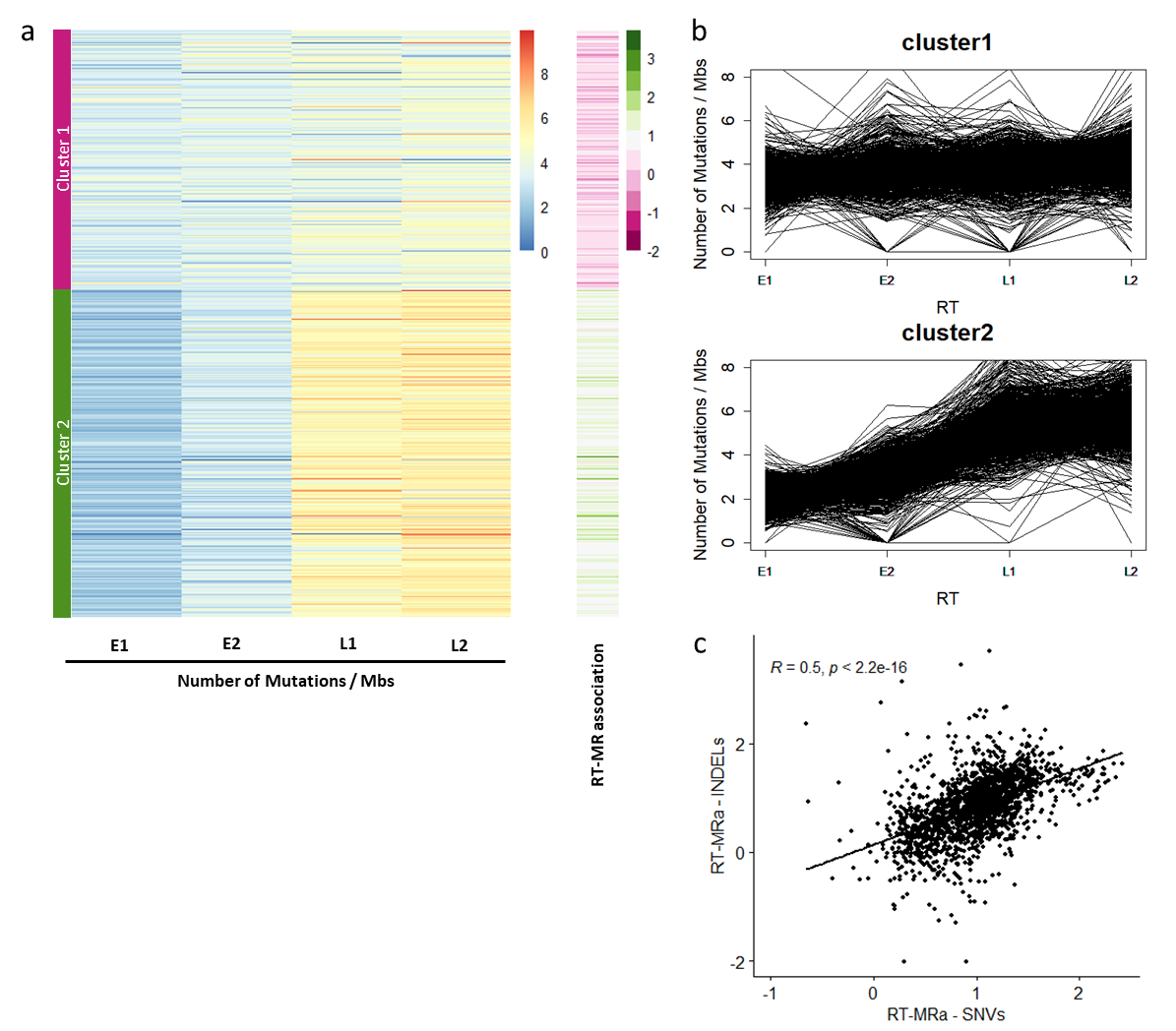


**Supplementary Figure S1. Uneven association between replication timing and mutation rates for Indels mutations**

(a) Heatmap capturing the mutation rate in each sample for four RT bins (E1-L2). The samples were clustered into two distinct clusters (using K-means, see **Methods**) with distinct association between RT and mutation rates. The right heatmap column captures the ratio of the mutation rates between early (E1+E2) and late (L1+L2) regions. (b) For each cluster, each line shows the mutation rate of a tumor sample at each RT region. (c) A scatter plot displaying the correlation between RT-MRa metric in SNVs and Indels mutations.

**Supplementary Figure S2**
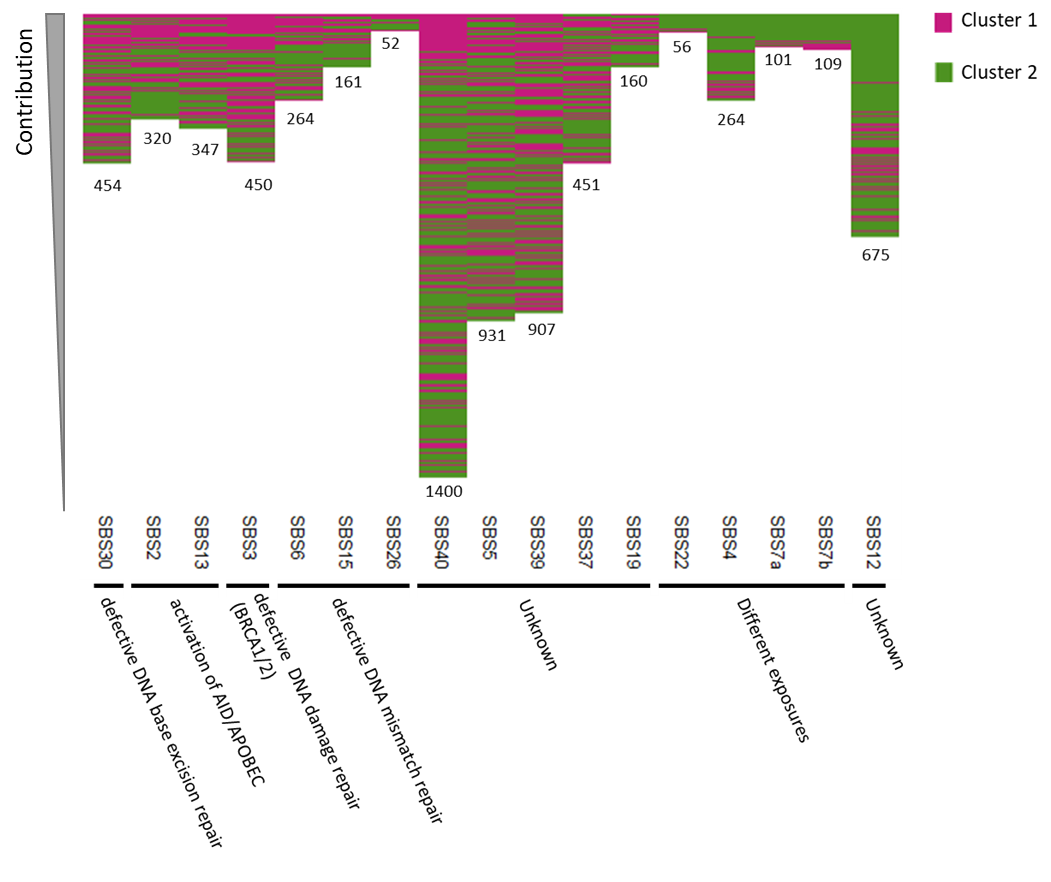


**Supplementary Figure S2. Association between RT-MR metric and Mutational signatures**

For each mutational signature the samples were sorted according to the relative contribution of the signature to the overall mutation load. Only samples in which the signature contribution is above 5% are shown. Samples belonging to cluster 1 are labeled in pink and samples from cluster 2 are green. Signatures with significant association between cluster assignment and contribution are shown. Only statistically significant signatures (adjusted P-val < 0.1; Kruskal-Wallis rank test). As indicated by the colors the left 11 signatures are enriched in cluster 1 samples, whereas the right 5 signatures are enriched in cluster 2 samples. Note that signatures enriched for cluster 1 are mainly associated with defects in DNA repair mechanisms.

**Supplementary Figure S3**
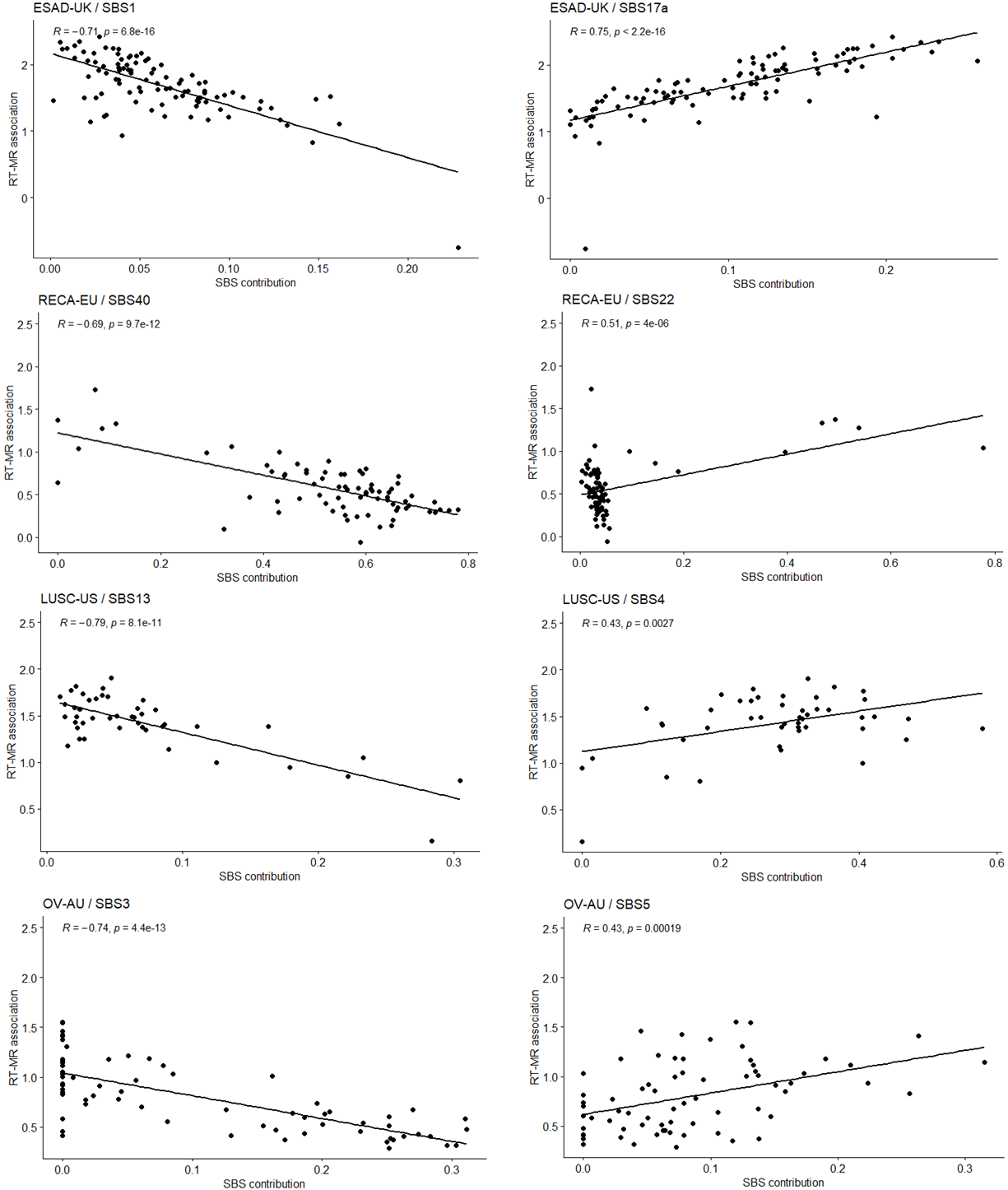


**Supplementary Figure S3. Association between RT-MR metric and Mutational signatures of specific projects**

Scatter-plots showing the association between RT-MR metric and signatures contribution for four projects; ESAD-UK, RECA-EU, LUSC-US and OV-AU. For each project two signatures are displayed – one with positive correlation (mark in red color in Fig. 2B) and one with negative correlation (mark in blue color in Fig. 2B).

**Supplementary Figure S4**

| 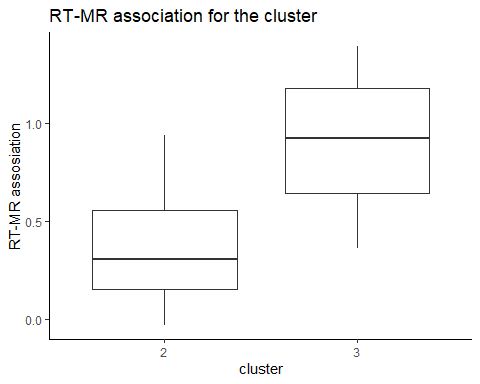 | 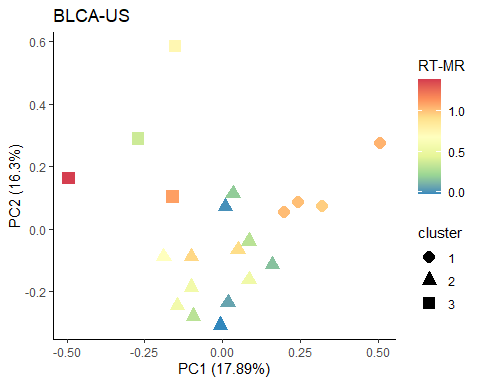 |
| --- | --- |
| 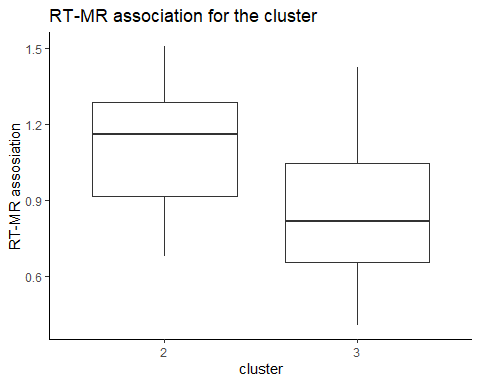 | 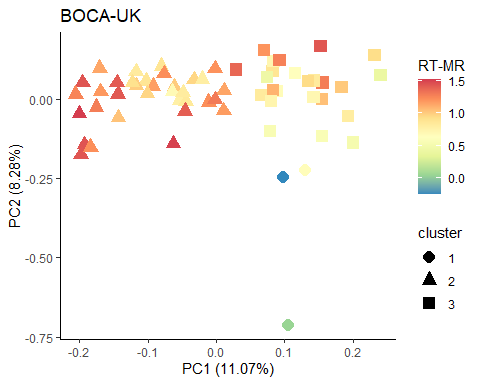 |
| 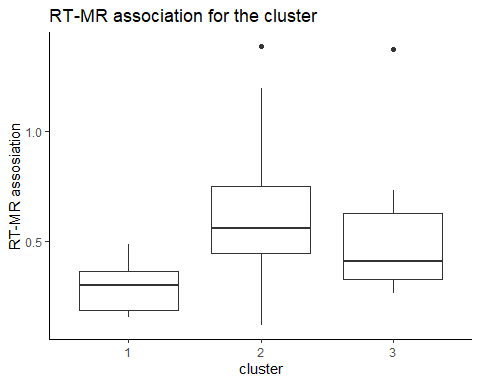 | 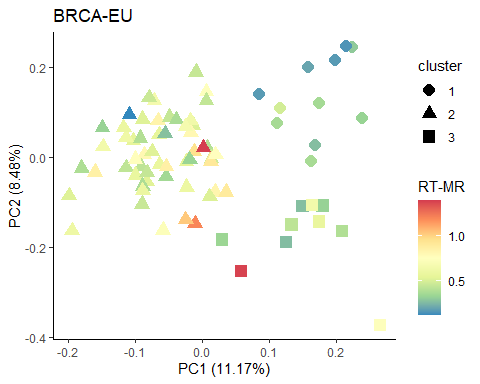 |
| 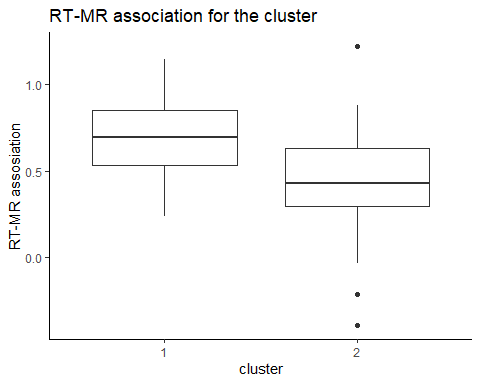 | 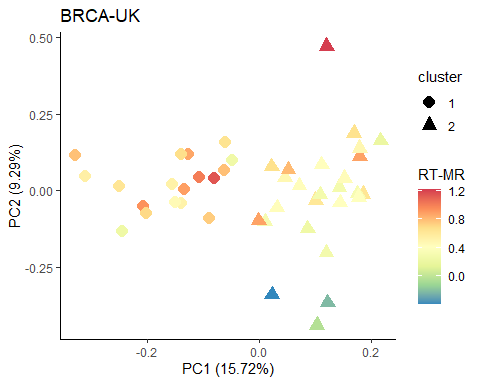 |
| 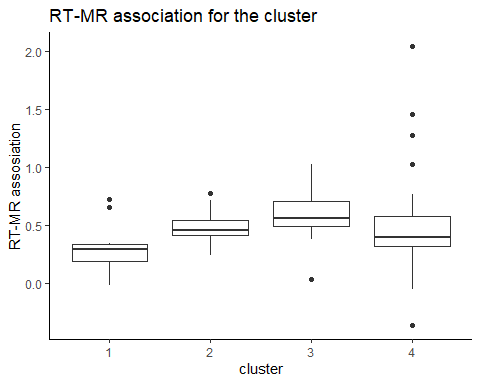 | 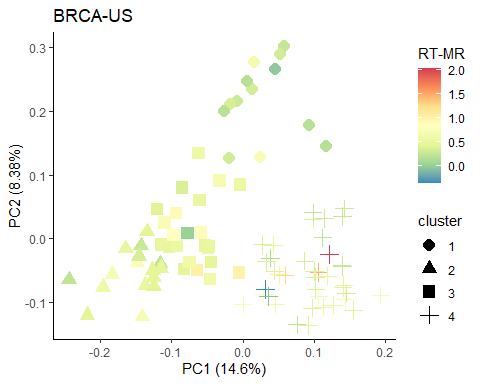 |
|  | 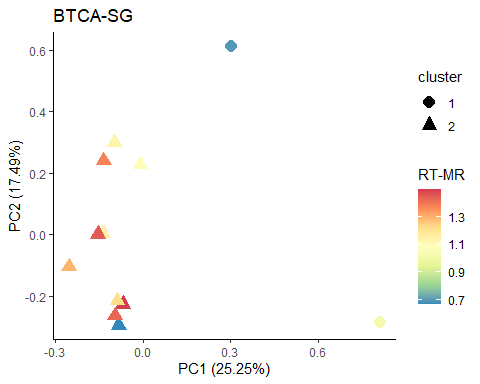 |
| 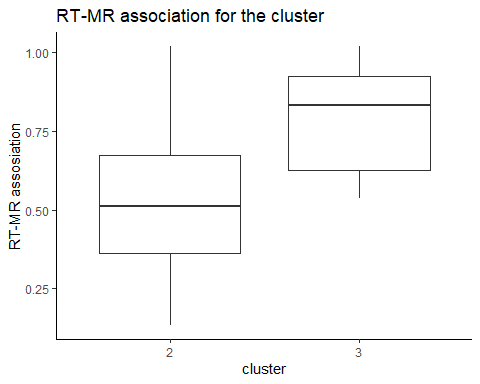 | 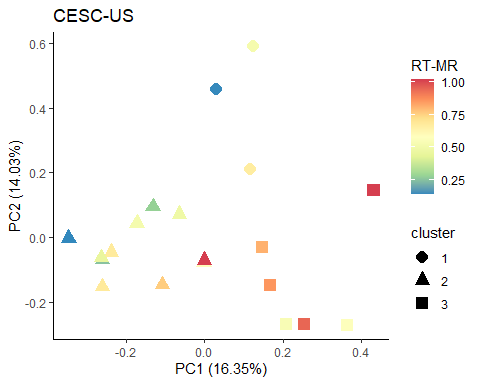 |
| 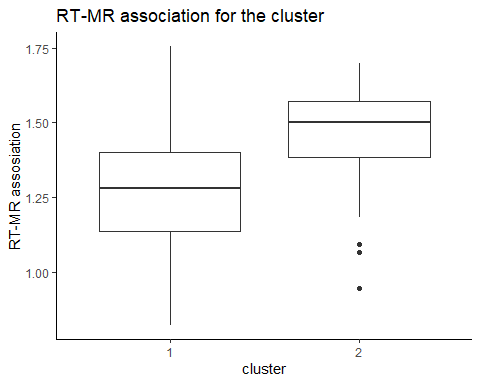 | 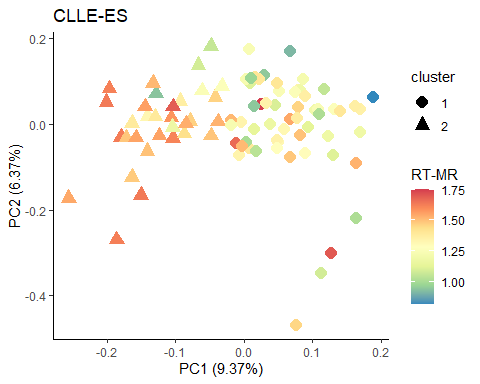 |
| 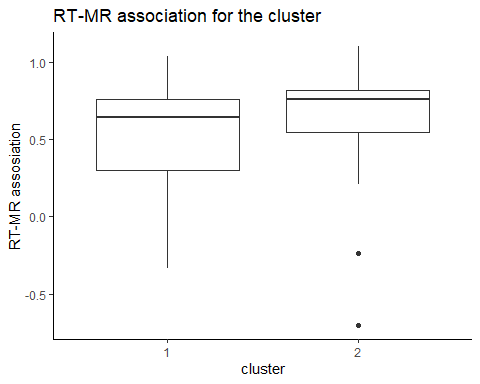 | 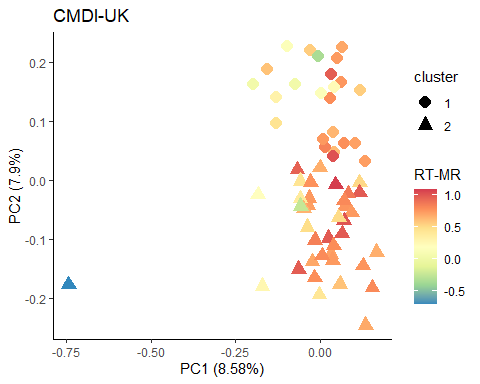 |
| 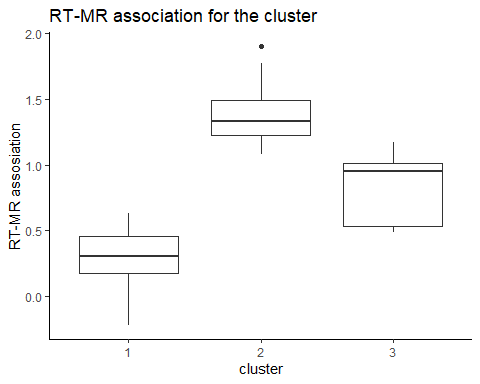 | 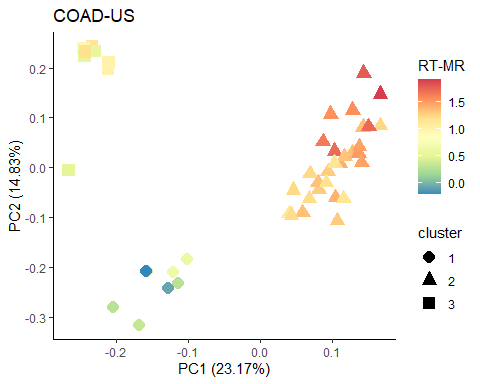 |
|  | 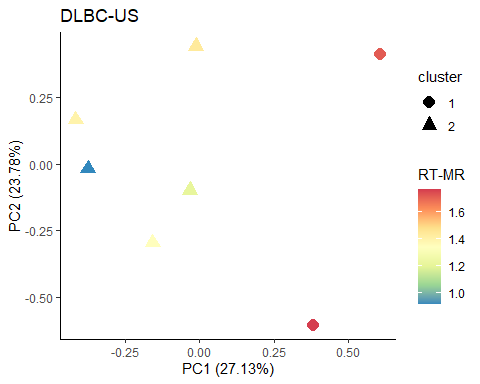 |
| 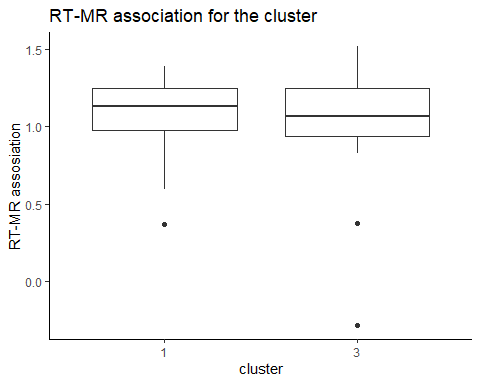 | 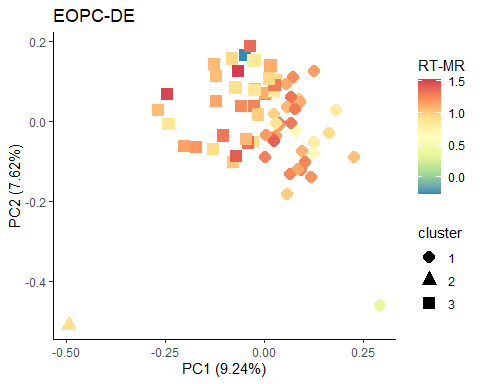 |
| 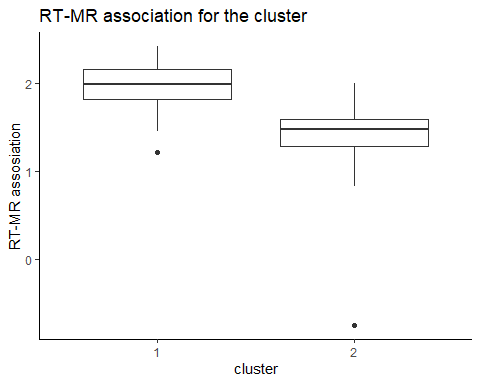 | 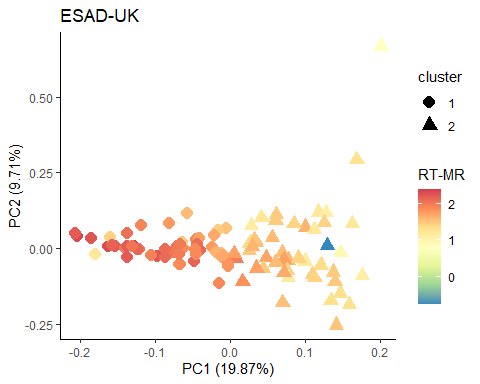 |
| 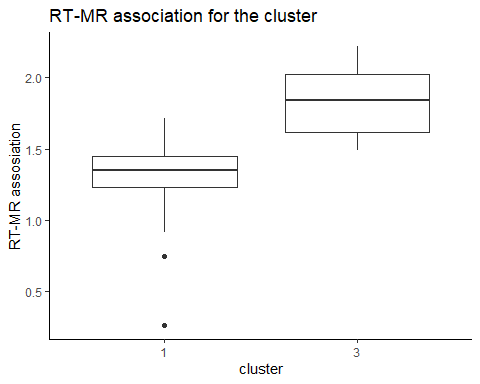 | 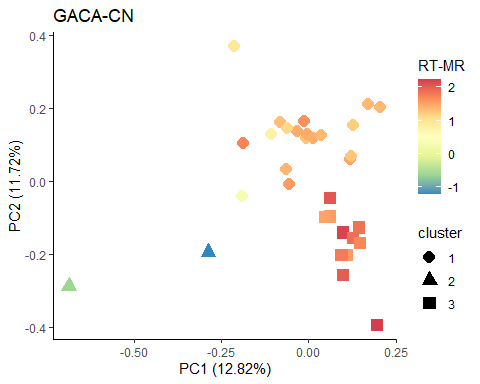 |
| 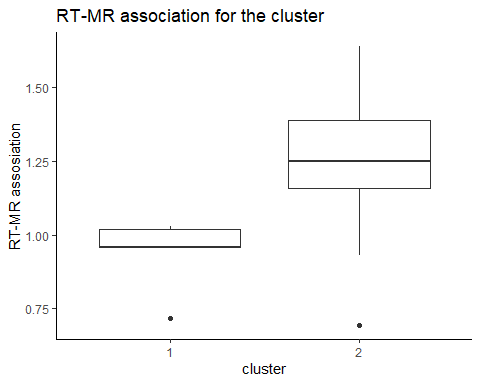 | 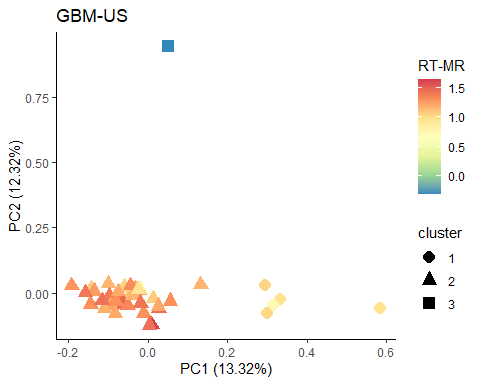 |
| 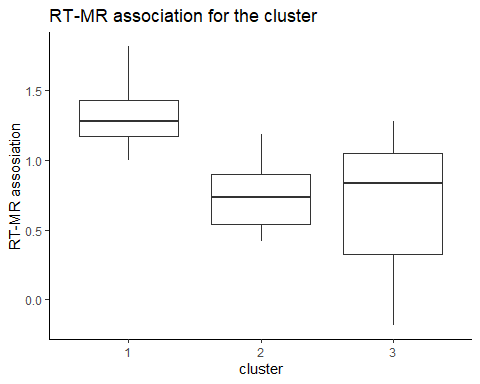 | 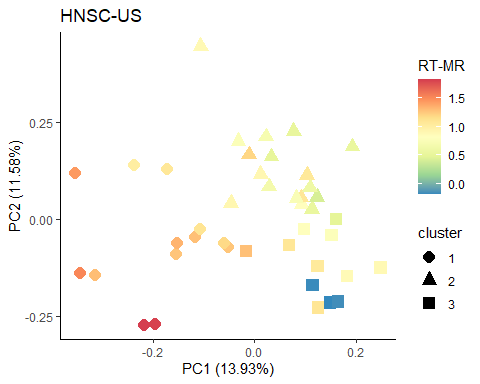 |
| 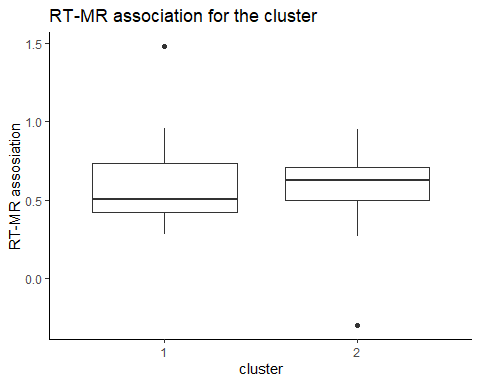 | 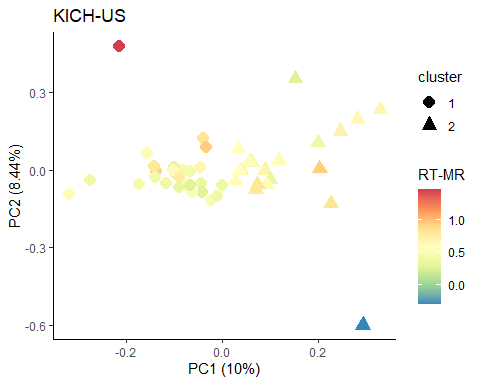 |
| 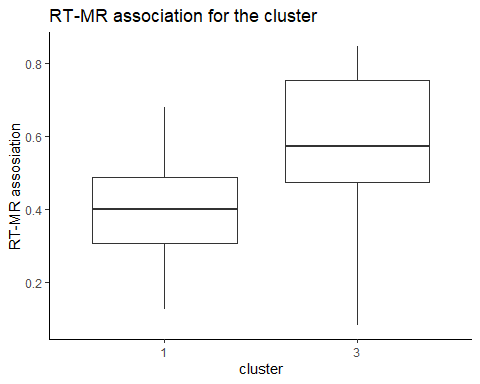 | 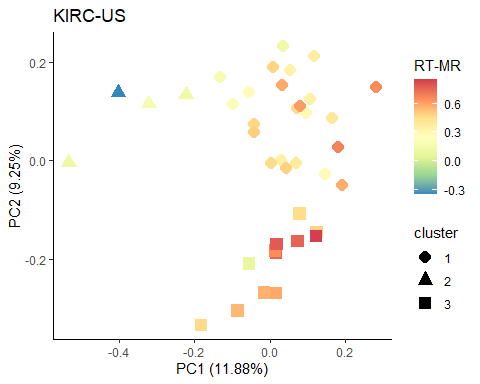 |
| 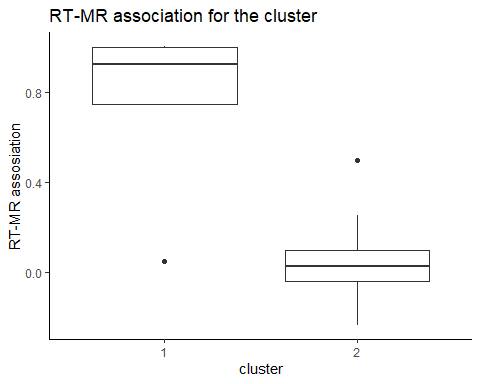 | 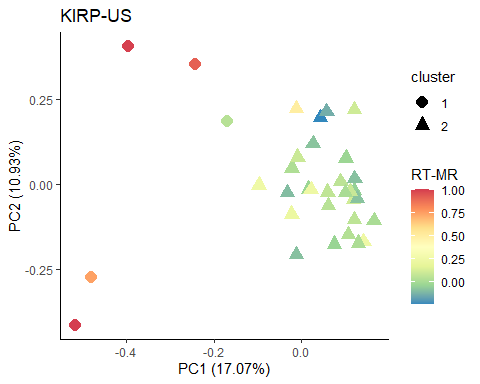 |
|  | 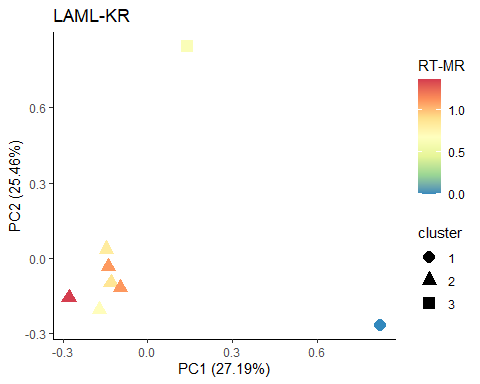 |
|  | 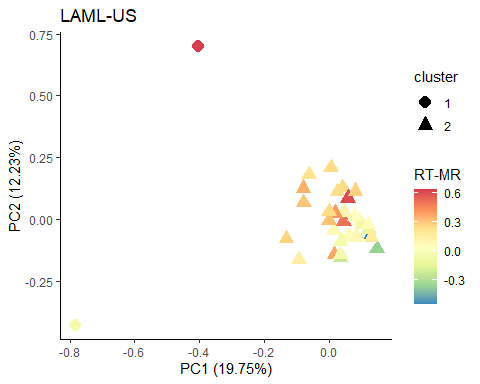 |
| 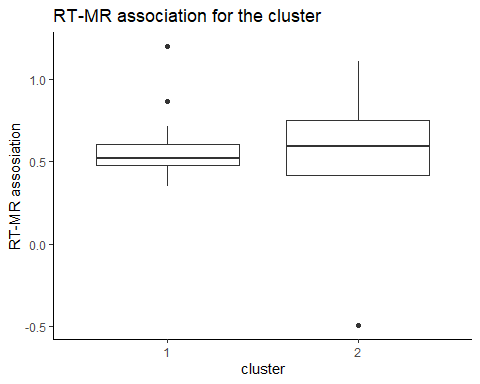 | 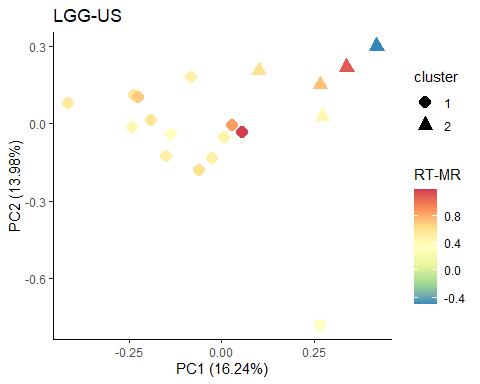 |
| 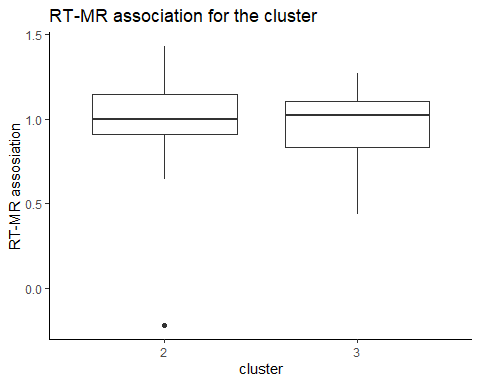 | 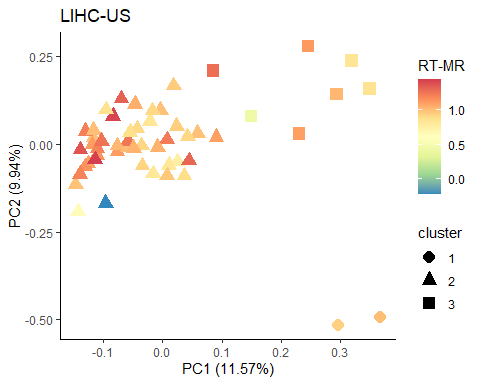 |
|  | 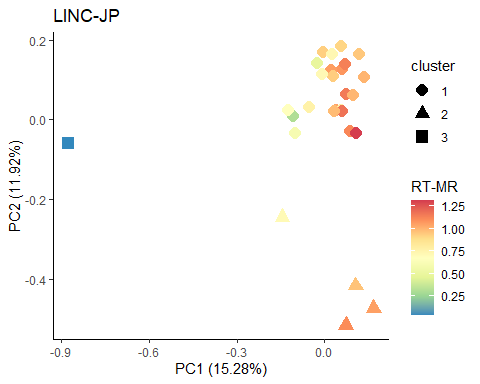 |
| 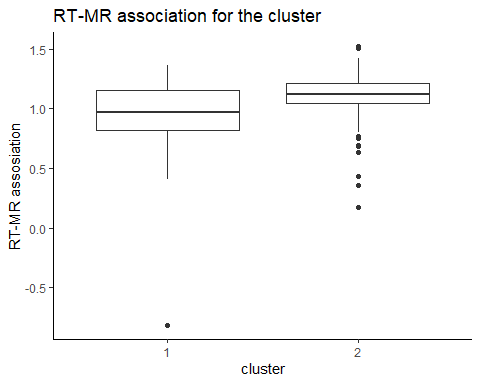 | 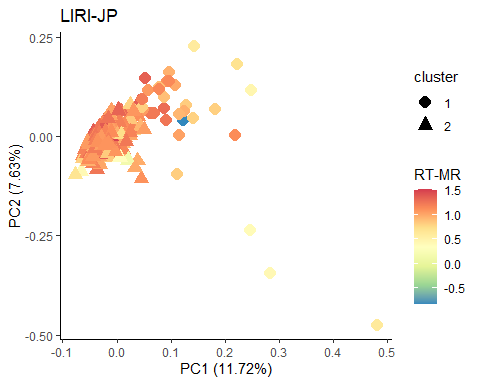 |
| 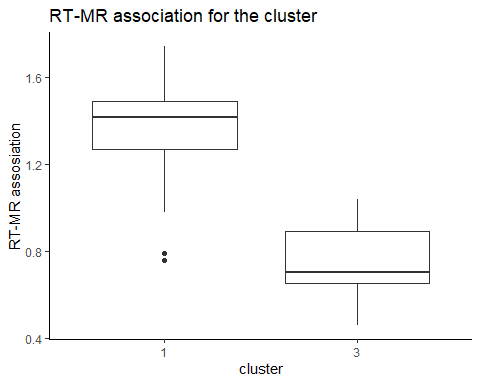 | 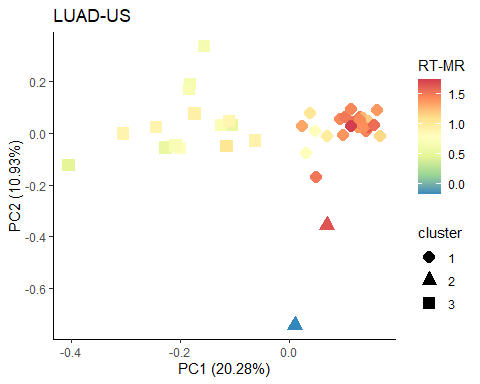 |
| 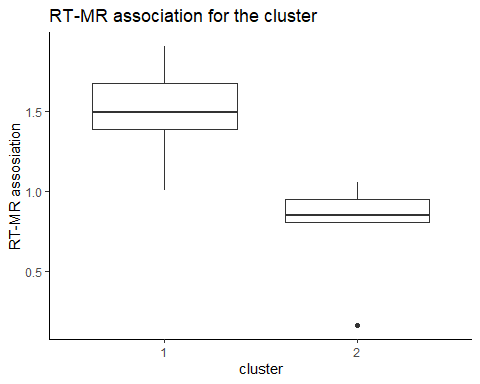 | 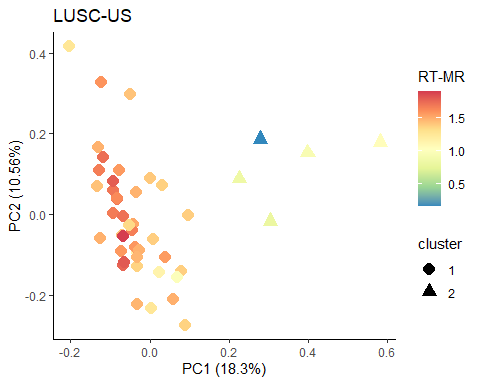 |
| 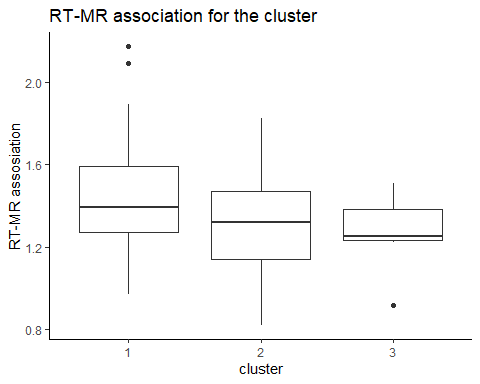 | 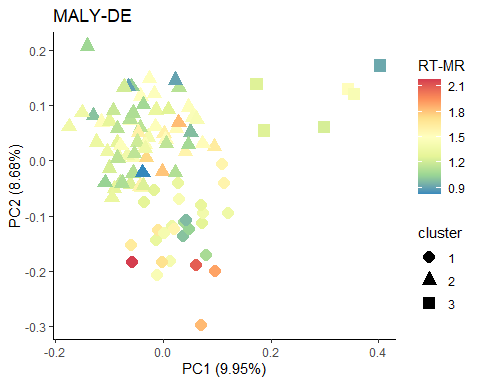 |
| 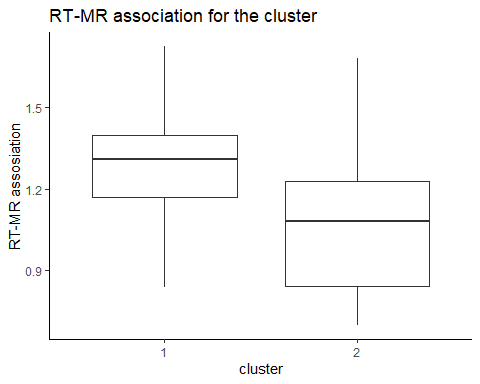 | 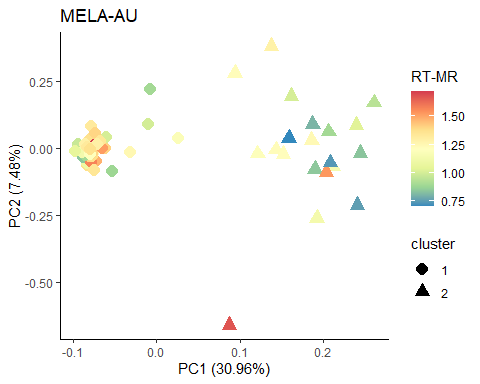 |
| 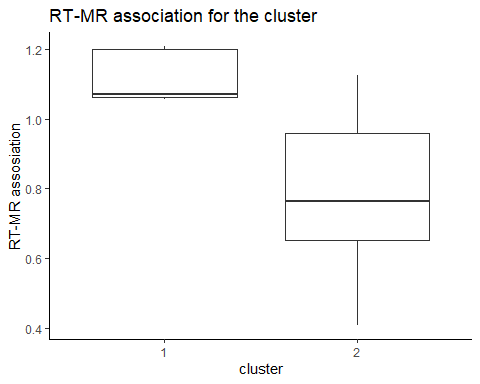 | 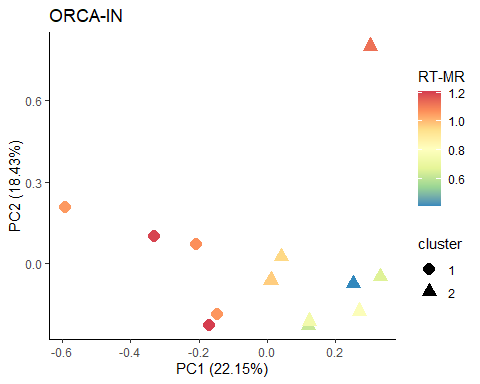 |
| 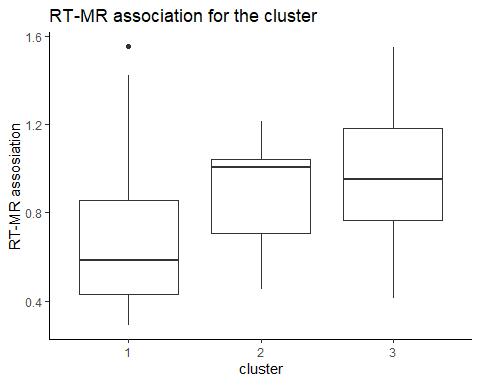 | 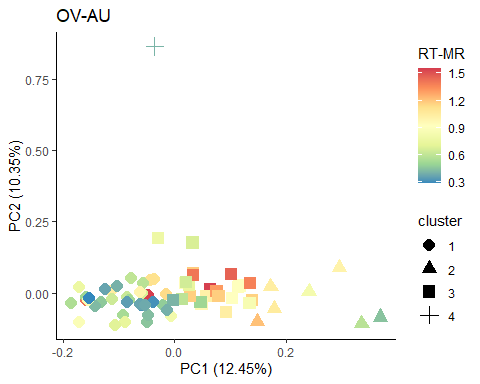 |
| 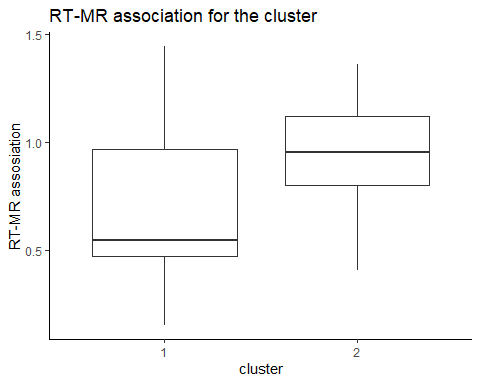 | 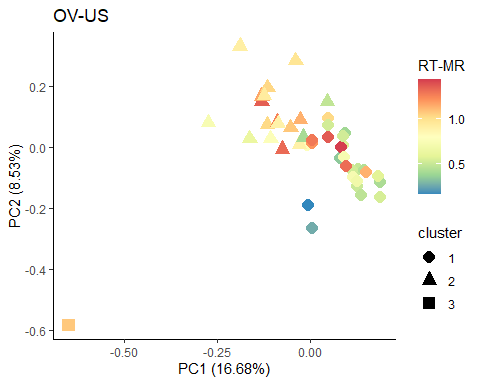 |
| 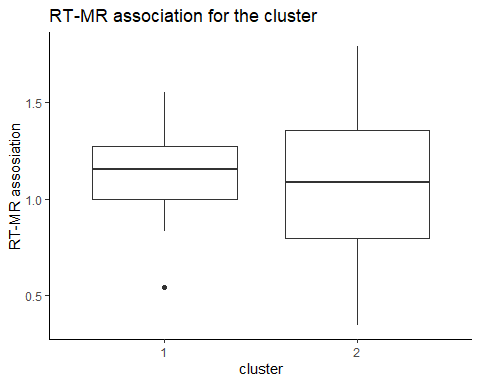 | 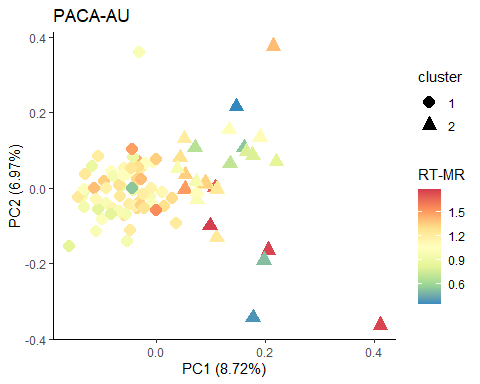 |
| 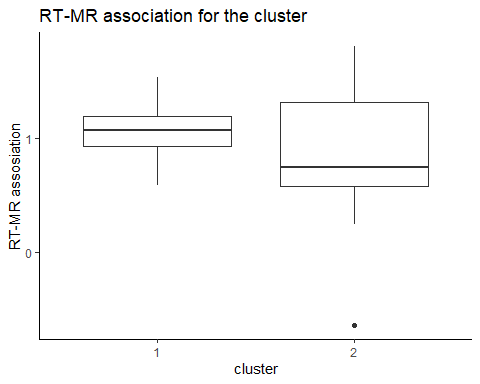 | 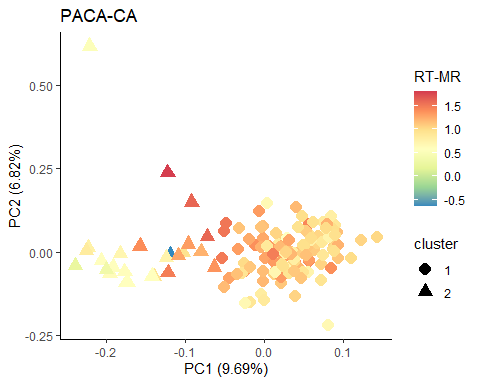 |
| 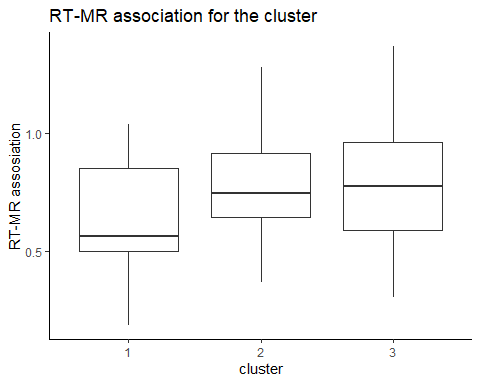 | 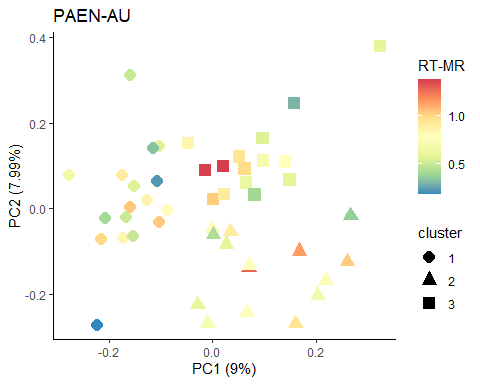 |
| 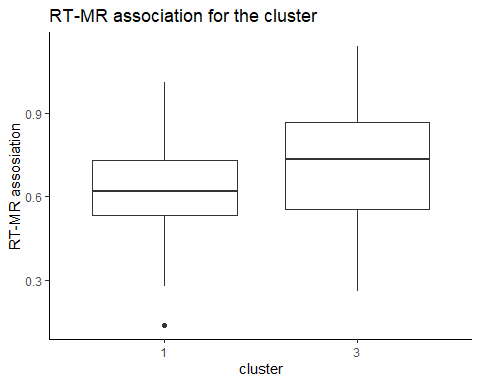 | 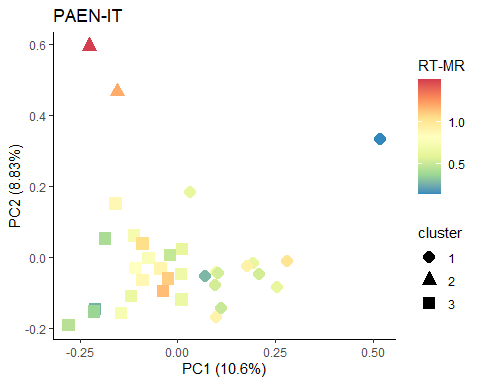 |
| 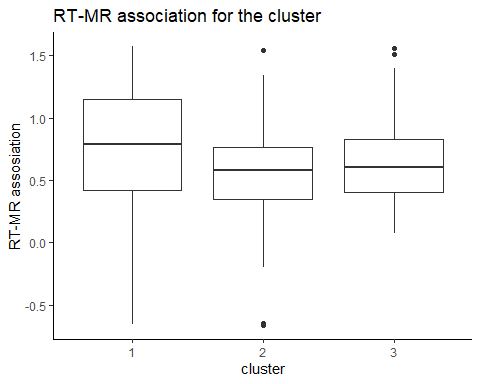 | 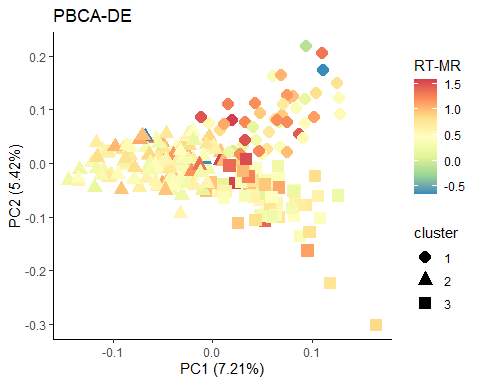 |
| 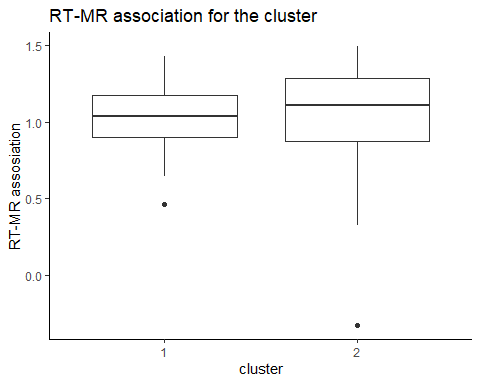 | 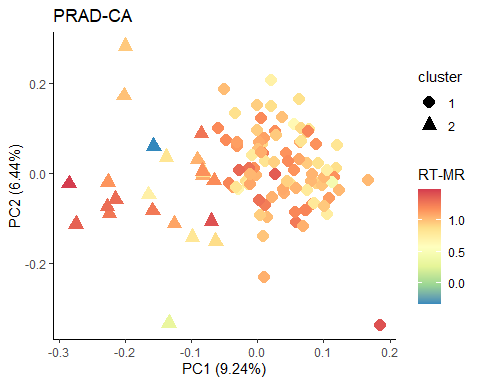 |
| 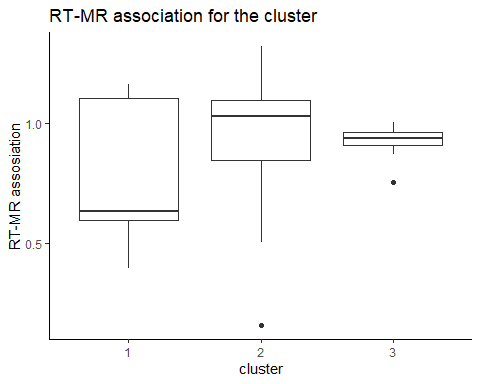 | 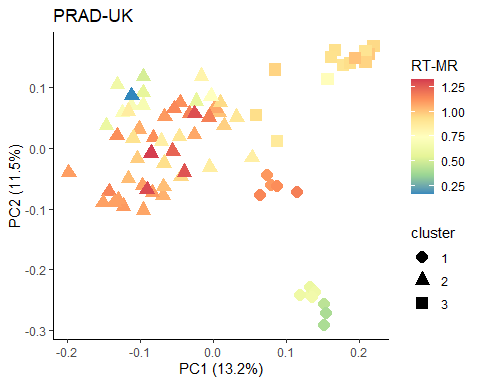 |
| 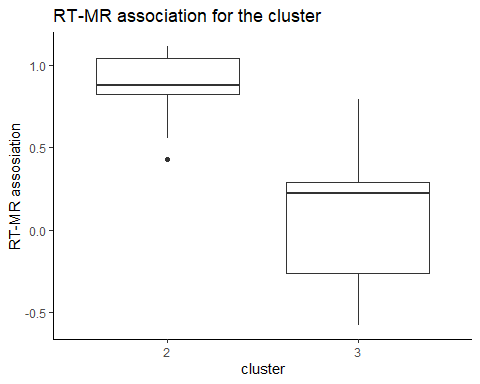 | 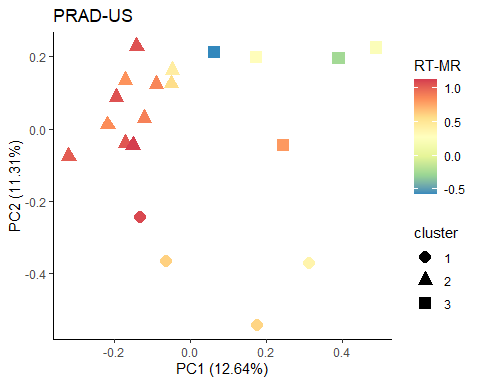 |
| 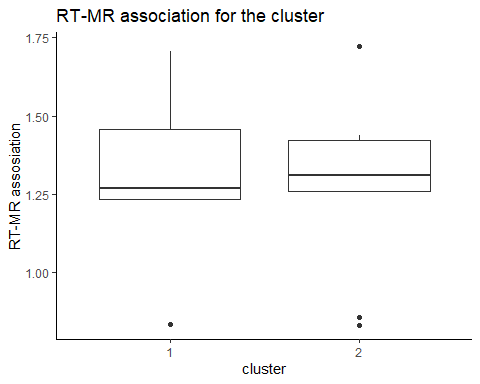 | 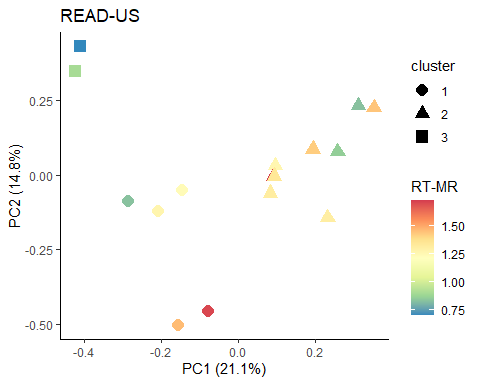 |
| 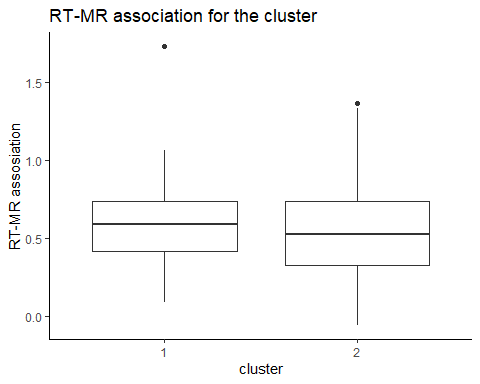 | 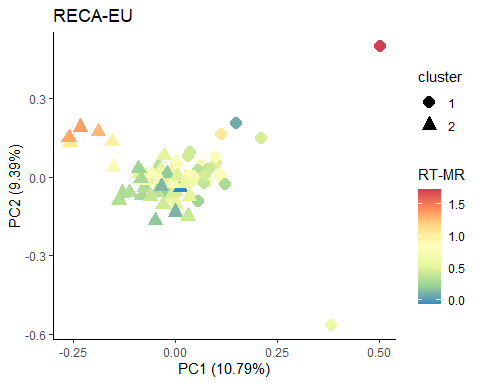 |
| 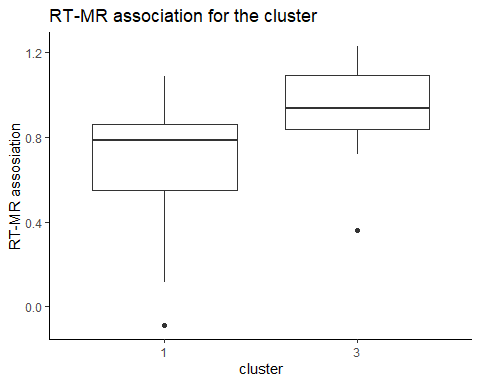 | 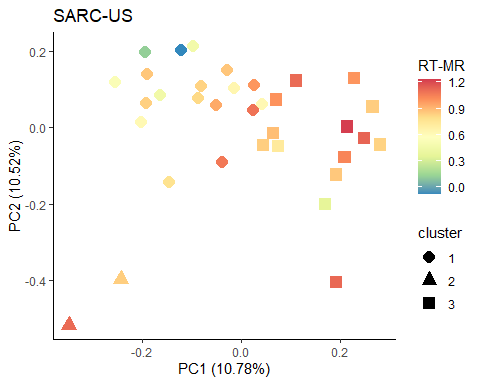 |
|  | 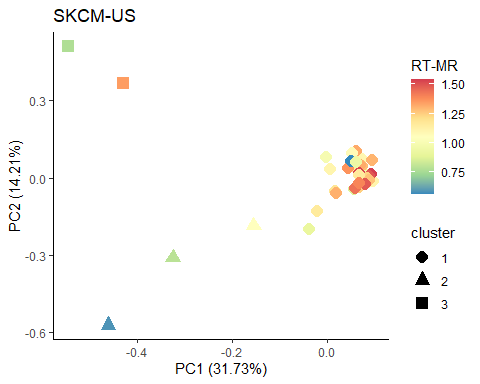 |
| 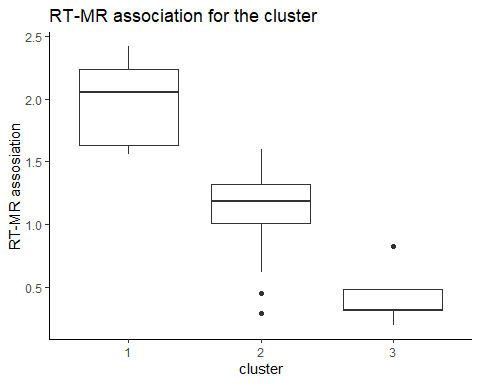 | 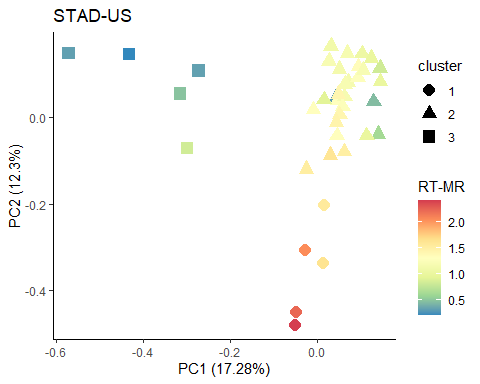 |
| 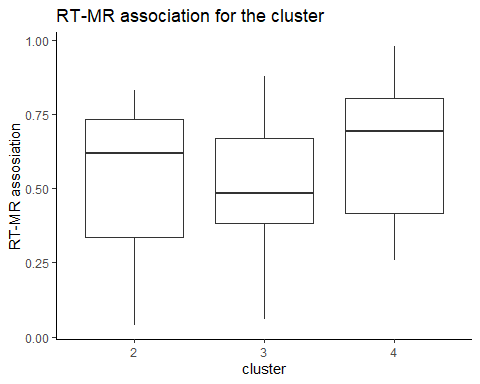 | 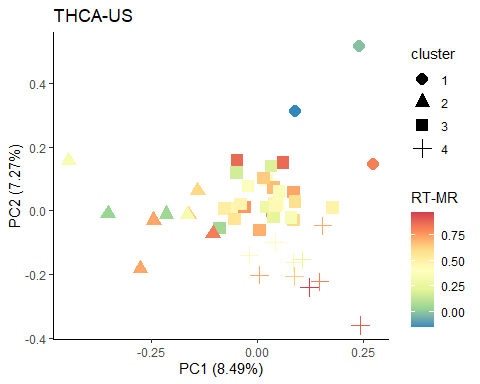 |
| 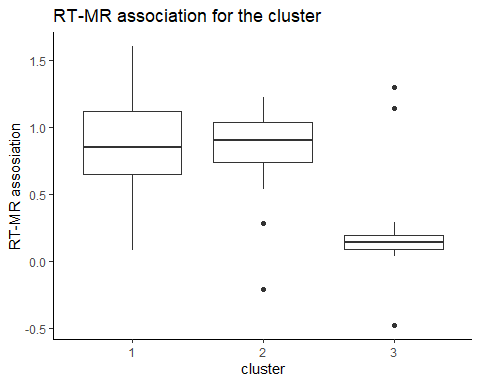 | 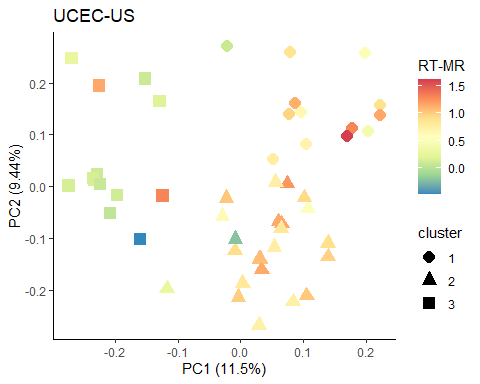 |

**Supplementary Figure S4. Contribution of combinations of signatures to the RT-MR associations**

For each project shown PCA plots of signature contribution data, colored by RT-MR association score and divided into different shapes by K mean clustering (left panel), and boxplot of the RT-MR association distribution of the different clusters (only for clusters with n>4). All P values derived from FDR-corrected Wilcoxon rank–sum test and shown in **Supplementary Table S1**.

**Supplementary Figure S5**


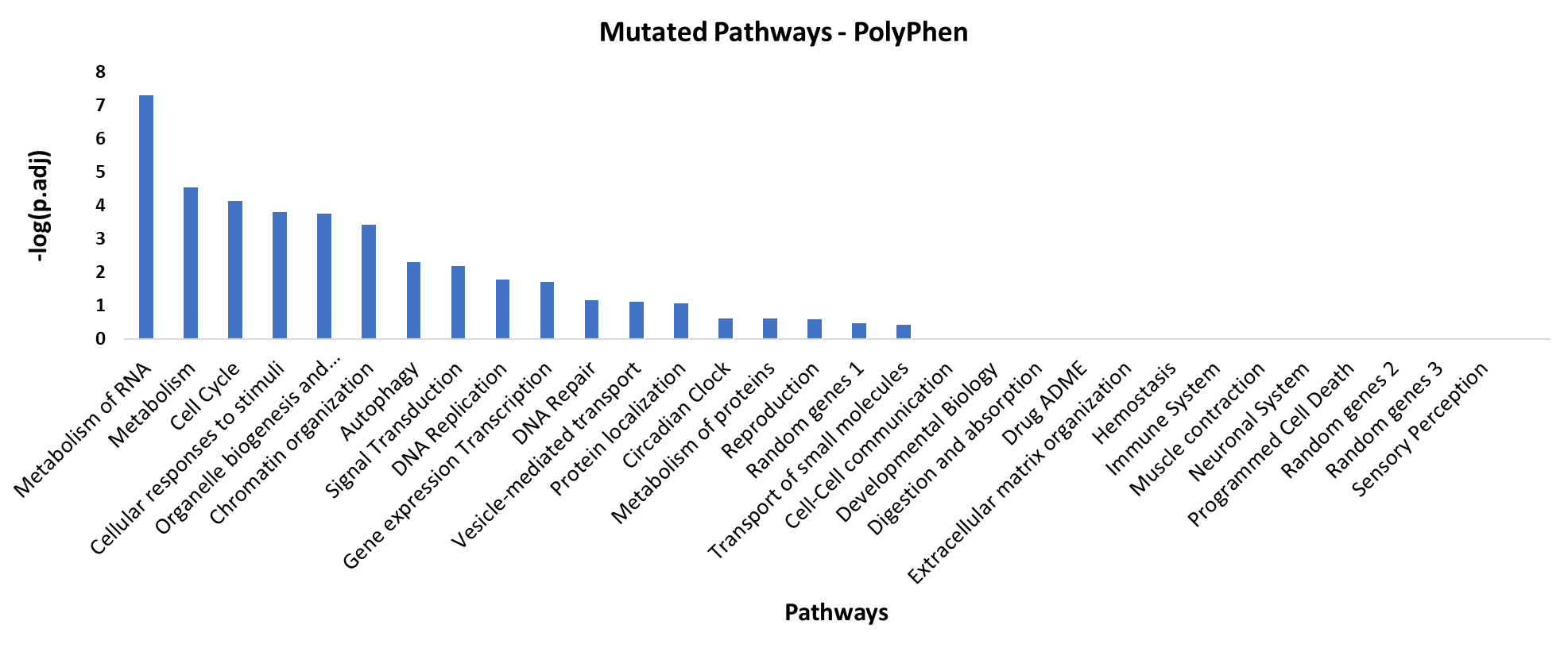


**Supplementary Figure S5. Mutated pathway analysis using different definition of deleterious genes**

Testing the different pathways for enrichment of deleterious mutations in the weak RT-MRa group. The deleterious mutation defined by the PolyPhen software as "probably damaging". Binomial tests were performed to test which pathways are enriched. The statistically significant enrichment pathways are those that pass the threshold of adjusted P-value < 0.1 (i.e. -log10(adjusted P-value) > 1).

**Supplementary Figure S6**
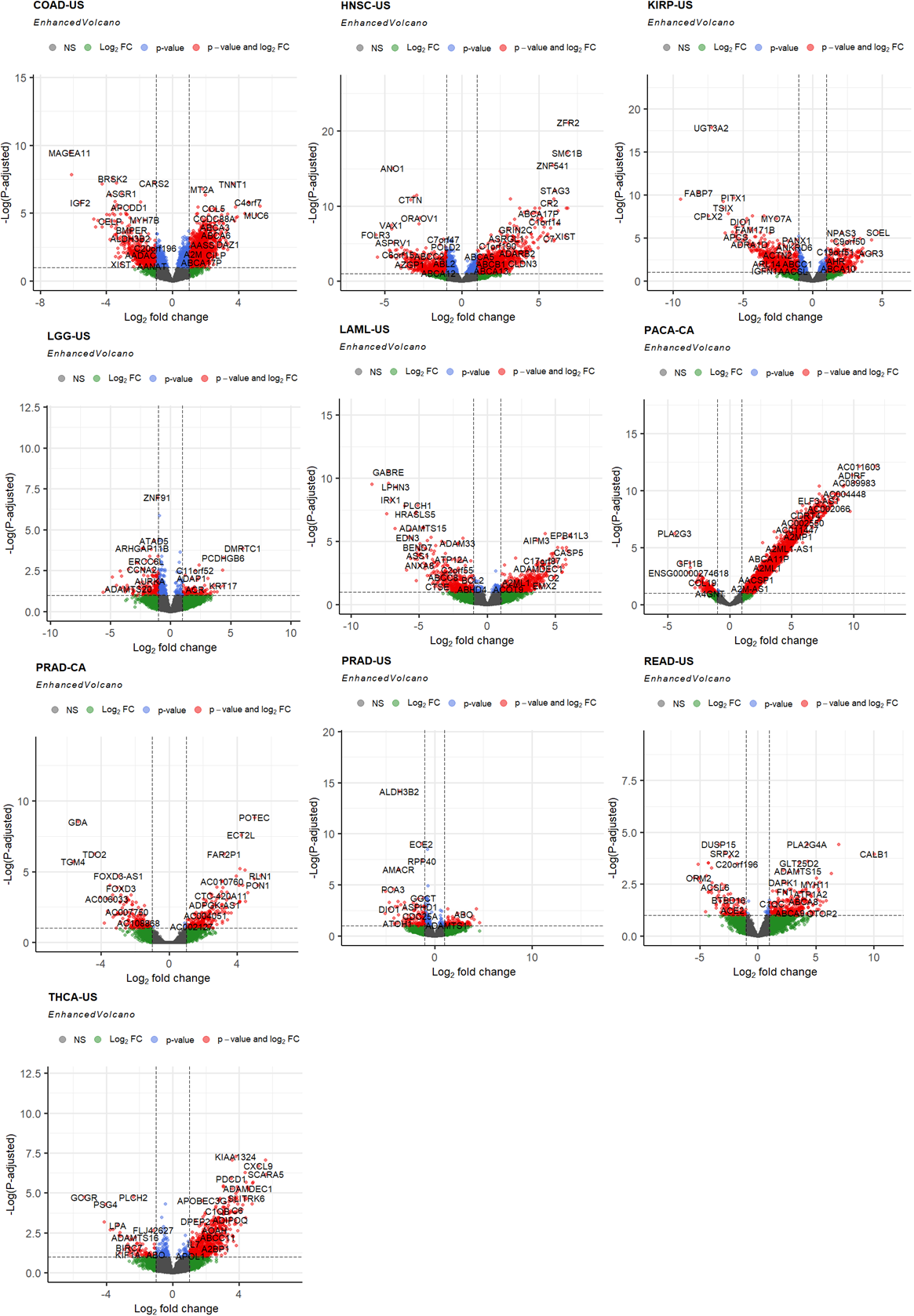


**Supplementary Figure S6. Expression profile analysis**

Volcano-plots of ten significant projects (projects in which the number of differentials expressed genes in the randomized data that passed the threshold was less than 10% of the number of genes identified by DESeq2). The red points are genes that are differentially expressed significantly between the low/high RT-MR groups. These points pass the thresholds of p.adjusted < 0.1 and log2FC >1.

**Supplementary Figure S7**


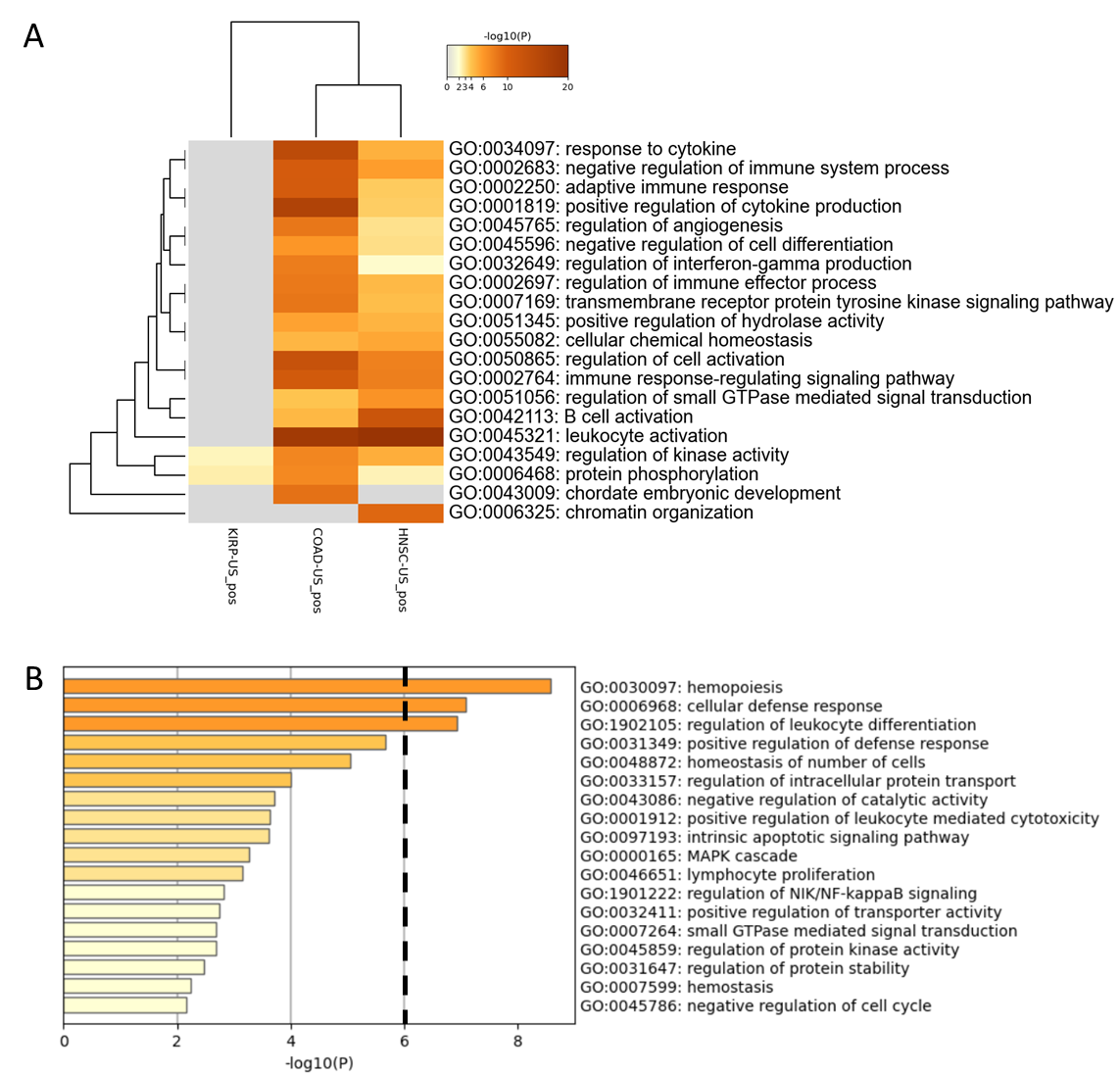


**Supplementary Figure S7. Expression profile analysis by Wilcoxon test of genes that expressed higher in the low RT-MR group**

(A) Heatmap capturing the significant enrichment of GO categories in the nine selected projects. The differential expression analysis performed by a Wilcoxon test rather by Deseq2 statistics. Non-significant enrichments (p.adjusted < 0.1) are colored grey. (B) GO analysis on the genes that appear in 2 projects. The GO categories are sorted according to the level of their significance. The vertical line at –log_10_(p)=6 designates the significance threshold after FDR correction.

**Supplementary Figure S8**

**
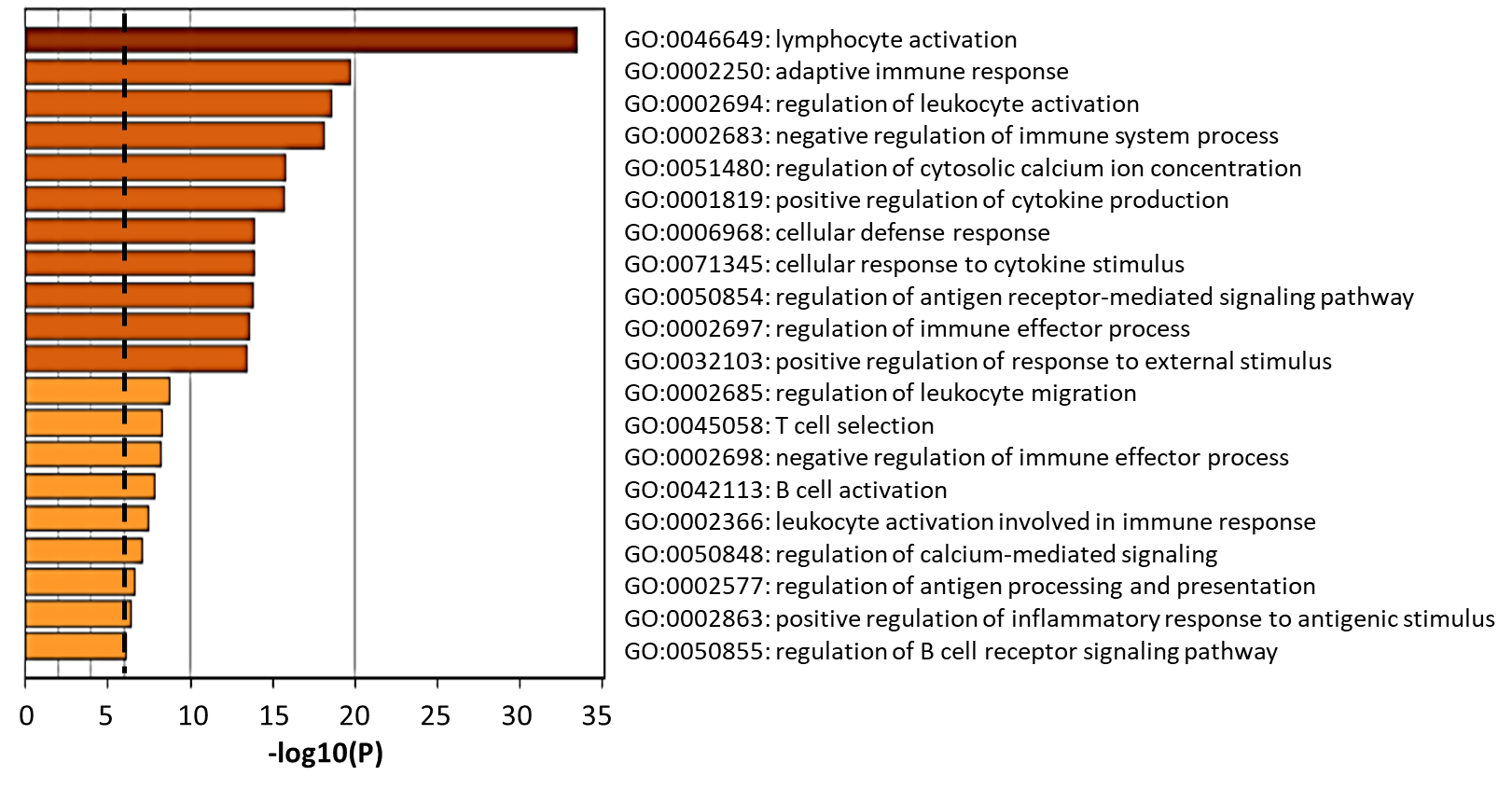
Supplementary Figure S8. Expression profile analysis**

GO analysis on the genes that appear in 3-5 projects. The GO categories are sorted according to the level of their significance. The vertical line at –log_10_(p)=6 designates the significance threshold after FDR correction.

**Supplementary Figure S9**


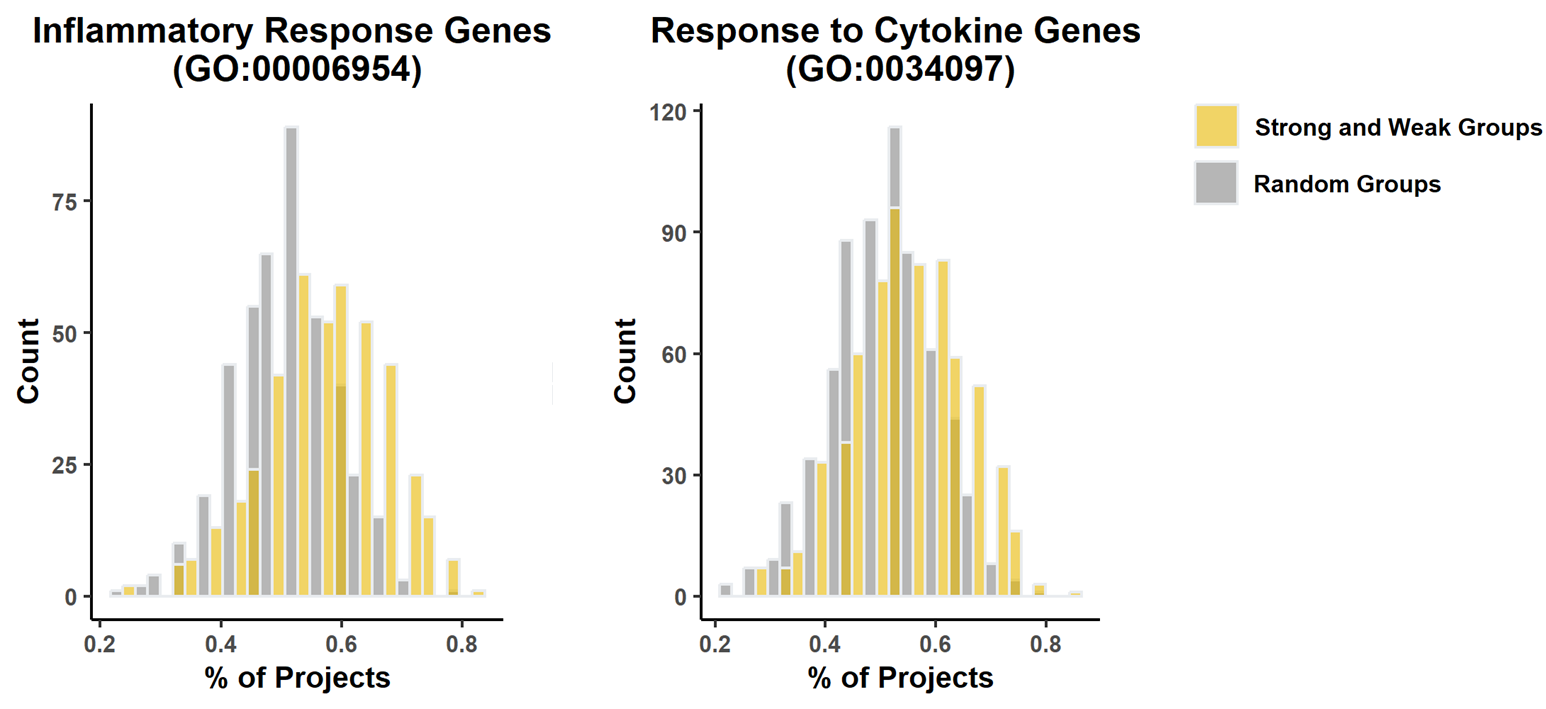


**Supplementary Figure S9. Immune related genes are expressed higher in the weak RT-MRa groups** Histograms capturing the bias in the expression of immune related genes to the weak RT-MRa group in multiple cancer types. For each gene we counted the percentage of projects it was enriched (logFC>0) in the weak RT-MRa group (orange bars). The distribution is skewed to the right in comparison to a random group of tumors (gray bars) (left panel: paired t-test, P val <10^-16^, effect size=0.79; right panel: P val <10^-16^, effect size=0.51).

**Supplementary Figure S10**


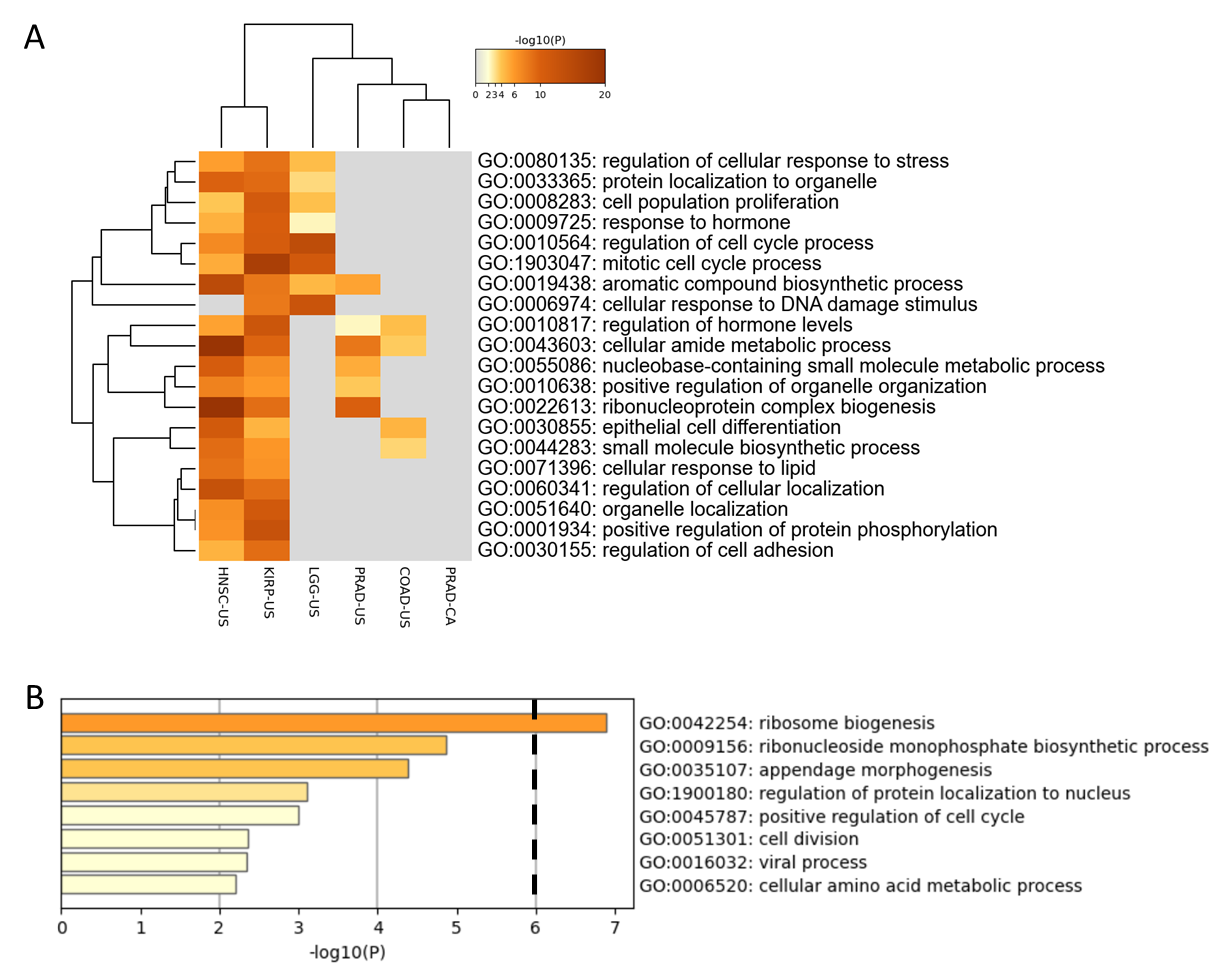


**Supplementary Figure S10. Expression profile analysis of genes that expressed higher in the high RT-MR group**

(A) Heatmap capturing the significant enrichment of GO categories in the nine selected projects for the complementary group of genes that are expressed higher in tumors with high RT-MR association. Non-significant enrichments (p.adjusted < 0.1) are colored grey. (B) GO analysis on the genes that appear in 2-4 projects. The GO categories are sorted according to the level of their significance. The vertical line at –log_10_(p)=6 designates the significance threshold after FDR correction.

**Supplementary Figure S11**

**
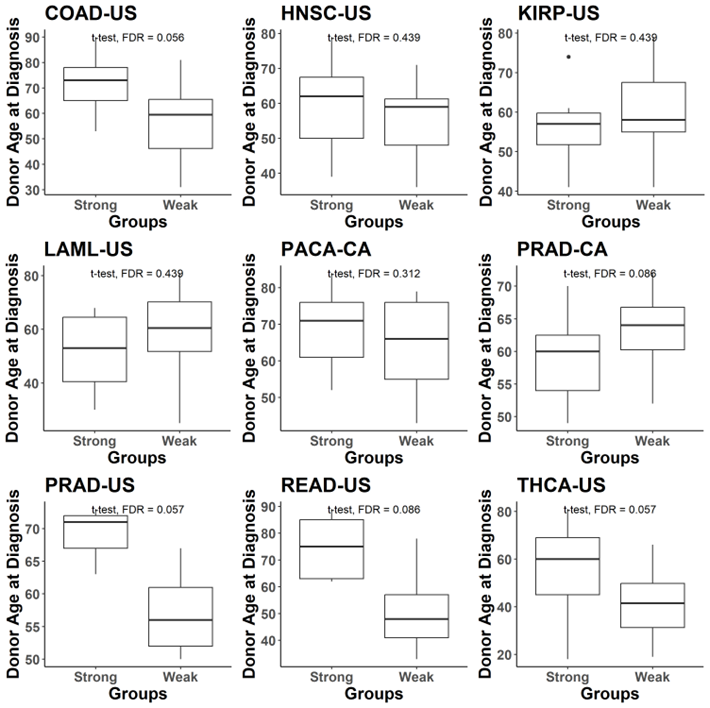
**

**Supplementary Figure S11. Donor age at diagnosis of the strong and weak groups**

Boxplot of the distribution of the donor age at diagnosis of the strong and weak groups in the different projects. All adjusted P values derived from t-test and Benjamini & Hochberg correction (FDR).

**Supplementary Figure S12**


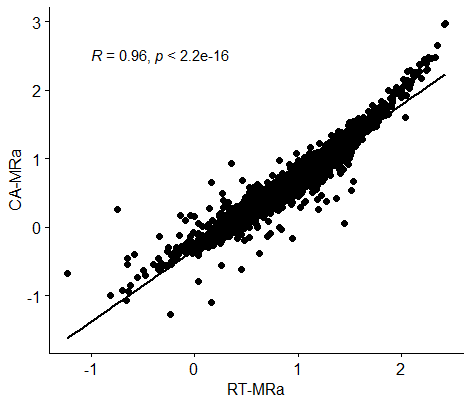


**Supplementary Figure S12. Correlation Between RT-MRa and chromatin states**

The scatter plots display correlation between RT-MRa metric and CA-MRa (chromatin accessibility - MR association metric) across the tumors (for the calculation of these metrics see **Methods**).

**Supplementary Figure S13**


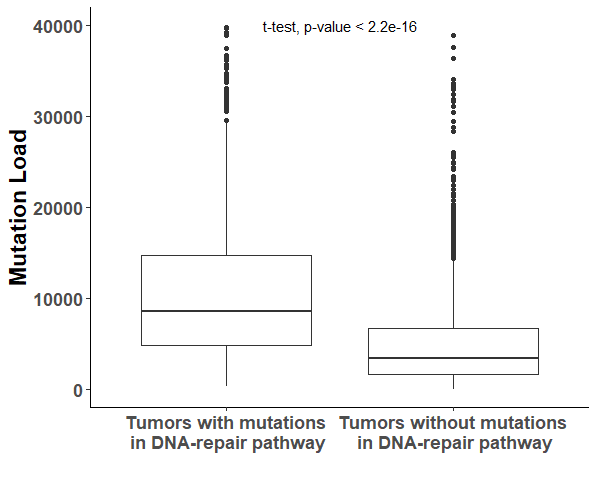


**Supplementary Figure S13. Mutations load in tumors with mutation in DNA repair pathway**

Boxplot of the distribution of mutations load in tumors with and without mutation in DNA repair pathway. P value derived from t-test.
