# Supplementary material for: Both cell autonomous and non-autonomous processes modulate the association between replication timing and mutation rate
Source: Sci Rep. 2023 Aug 12;13:13143. doi: 10.1038/s41598-023-39463-1 (PMC10423235; doi:10.1038/s41598-023-39463-1)
Supplement: Supplementary file 2 — Supplementary Figures. [file 41598_2023_39463_MOESM2_ESM.docx]

**Legends for supplementary tables**

**Supplementary Table S1**

Association of subgroups of signatures contribution with the RT-MRa metric using Wilcoxon rank sum test. The subgroups of tumors within each project defined used principal component analysis (PCA) followed by K-means clustering (see Methods).

**Supplementary Table S2**

List of enriched mutated genes. For each enriched pathway – all included genes tested individually. A one-sided binomial test examined which mutated gene is enriched in cluster 1.

**Supplementary Table S3**

The file "Evidence.csv" from Metascape analysis of the Expression profile analysis of genes that expressed higher in the low RT-MR group.

**Supplementary Table S4**

The file "FINAL_GO.csv" from Metascape analysis of the Expression profile analysis of genes that expressed higher in the low RT-MR group.

**Supplementary Table S5**

The file "Evidence.csv" from Metascape analysis of the Expression profile analysis by Wilcoxon test of genes that expressed higher in the low RT-MR group

**Supplementary Table S6**

The file "FINAL_GO.csv" from Metascape analysis of the Expression profile analysis by Wilcoxon test of genes that expressed higher in the low RT-MR group

**Supplementary Table S7**

The file "Evidence.csv" from Metascape analysis of the Expression profile analysis of genes that expressed higher in the high RT-MR group.

**Supplementary Table S8**

The file "FINAL_GO.csv" from Metascape analysis of the Expression profile analysis of genes that expressed higher in the high RT-MR group.
